# Evaluating Artificial Intelligence, Peer, and Instructor Feedback Across Scientific Communication Assignments in Undergraduate Biology

**DOI:** 10.1002/ece3.74365

**Published:** 2026-09-20

**Authors:** Lori Boies

**Affiliations:** ^1^ St. Mary's University San Antonio Texas USA

**Keywords:** artificial intelligence feedback, formative feedback, science communication, undergraduate biology education, writing‐to‐learn

## Abstract

Generative artificial intelligence (AI) has emerged as a potential source of formative feedback for student writing. However, relatively little is known about how AI‐generated feedback compares with instructor and peer feedback across different Writing‐to‐Learn (WTL) assignments implemented in authentic undergraduate biology classrooms. This exploratory study compared AI (specifically ChatGPT‐5), peer, and instructor feedback across infographic and academic explanatory article assignments implemented in three undergraduate biology courses representing diverse student populations. Agreement among feedback sources was evaluated using intraclass correlation coefficients (ICC), while student perceptions of feedback usefulness, trust, engagement, and effort were assessed using Likert‐scale survey data. Agreement between AI and instructor feedback varied according to assignment modality, disciplinary context, and revision stage. AI demonstrated moderate agreement with instructor feedback during the draft stage, but agreement frequently diverged following student revision, particularly for higher‐order rubric criteria such as content accuracy and disciplinary reasoning. Agreement was consistently stronger for academic explanatory articles than for infographics. Students perceived instructor feedback as more useful and trustworthy than either peer or AI feedback, whereas the effort required to apply feedback did not differ across feedback sources. AI feedback was generally perceived similarly to peer feedback. Collectively, these findings suggest that AI is best used as a formative, early‐stage scaffold within WTL assignments rather than as a summative evaluation tool. When integrated into structured instructional workflows alongside instructor and peer feedback, large language models can support student revision while preserving the central role of instructor expertise in undergraduate biology education.

## Graphical abstract

This study examined how AI (ChatGPT‐5), peer, and instructor feedback compare across Writing‐to‐Learn assignments in undergraduate biology. AI‐generated feedback demonstrated moderate agreement with instructor evaluations and was perceived similarly to peer feedback, supporting its role as a formative tool that complements, rather than replaces, instructor expertise.

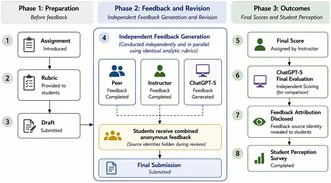

## 1 Introduction

Formative feedback is a cornerstone of effective writing instruction, enabling students to refine their ideas, strengthen disciplinary understanding, and develop scientific communication skills through iterative revision (Jani and Mellinger 2015; Giles et al. 2013; Hattie and Timperley 2007; Geithner and Pollastro 2016). Writing‐intensive assignments have become an important component of undergraduate biology education because they cultivate skills beyond simple recall of course content, including the ability to critically engage with scientific knowledge, apply concepts in novel contexts, and communicate scientific information effectively. Within the sciences, there has been a sustained call to action to strengthen students' training in science communication as an integral component of undergraduate education (Brownell et al. 2013a, 2013b; Finkenstaedt‐Quinn et al. 2022; Yore et al. 2004; Laakko Train and Miyamoto 2017; Mercer‐Mapstone and Kuchel 2016). However, the educational value of these assignments depends on students receiving timely, specific, and actionable feedback; a process that can be both labor‐intensive and difficult to scale (Giles et al. 2013; Hattie and Timperley 2007). As instructors increasingly seek approaches that maintain feedback quality while reducing workload, generative artificial intelligence (AI) offers the potential to augment established feedback practices.

Effective feedback that is specific, actionable, and targeted has been shown to positively influence the student learning experience and effectively address knowledge gaps (Jani and Mellinger 2015; Giles et al. 2013; Hattie and Timperley 2007). Students who receive feedback from multiple sources, including instructors and peers, generally produce stronger written work and demonstrate a deeper understanding of the underlying content (Cho and MacArthur 2010; Cho and Schunn 2007; Finkenstaedt‐Quinn et al. 2024). Although peer feedback is an established pedagogical strategy that can enhance scientific writing and literacy, the consistency and inter‐rater agreement of peer‐generated evaluations can be highly variable (Geithner and Pollastro 2016; Falchikov and Goldfinch 2000). These limitations have prompted interest in additional sources of structured formative feedback that can complement, rather than replace, the established dyad of instructor and peer feedback practices. The advent of large language model (LLM)–based artificial intelligence (AI) technologies, such as ChatGPT, has generated considerable interest in leveraging these tools to provide objective, scalable feedback to students across Writing‐to‐Learn (WTL) assignments such as an infographic or academic explanatory article (Liew and Tan 2025). Existing studies have reported mixed findings regarding students' perceptions of the usefulness of AI feedback, with most research conducted in disciplines such as math and computer science (Shin et al. 2025; Er et al. 2025; Tossell et al., n.d.). AI‐generated feedback should be viewed as a formative support capable of identifying broad opportunities for revision, while instructor feedback remains essential for addressing discipline and course‐specific content and assignment‐specific nuances (Er et al. 2025; Tossell et al., n.d.; Drinkwater Gregg et al. 2025; Holmes et al. 2019; Pan 2025). Although previous studies have demonstrated the potential utility of AI‐generated feedback, most have focused on single courses, single assignment types, or student perceptions alone, leaving unanswered questions regarding the consistency of AI‐generated rubric scoring relative to instructor and peer feedback in authentic classroom settings (Shin et al. 2025; Er et al. 2025; Tossell et al., n.d.; Drinkwater Gregg et al. 2025; Holmes et al. 2019; Henderson et al. 2026).

Despite increasing interest in AI‐supported formative feedback, relatively few studies have simultaneously compared instructor, peer, and AI‐generated feedback across undergraduate courses and WTL assignments while also examining student perceptions of each feedback source. Furthermore, little is known about whether agreement among feedback providers differs across common biology writing assignments implemented within routine classroom practice. This exploratory study examined two research questions. First, to what extent did rubric scores assigned by AI (ChatGPT‐5), peers, and the instructor demonstrate agreement across undergraduate biology writing assignments? Second, how did students perceive the usefulness, trustworthiness, engagement, and effort associated with instructor, peer, and AI‐generated feedback when the source of the feedback was known? To address these questions, inter‐rater reliability analyzes were utilized to compare rubric scores among feedback providers, and Likert‐scale survey data were analyzed to characterize student perceptions of each identified feedback source. By integrating measures of inter‐rater agreement with student perceptions, this study provides an evidence‐based evaluation of AI‐generated feedback within authentic classroom contexts. These findings contribute to ongoing discussions in STEM education regarding the responsible integration of AI tools (such as LLMs) into existing feedback workflows while offering practical guidance for instructors seeking support and scalable approaches to WTL assignments.

## 2 Methods

### 2.1 Study Context and Participants

This study was conducted during the Fall 2025 semester at St. Mary's University (StMU), a private, Catholic, Hispanic‐serving institution located in San Antonio, Texas. The project was classified as program evaluation and determined to be not regulated research by the StMU Institutional Review Board. Students who elected to participate in the post‐study survey were provided informed consent prior to inclusion of their deidentified responses.

Data were collected across three undergraduate biology courses representing differing levels of disciplinary complexity: BL 2311 Food and Nutrition (non‐majors; enrollment = 45), BIO 3310 Introduction to Bioinformatics (majors; enrollment = 17), and BL 4411 Genes, Genomes, and Genomics (majors; enrollment = 27). The author was the sole instructor for these courses and was responsible for design, instruction, and assessment.

### 2.2 Assignment Design and Feedback Procedures

Two WTL assignment genres were examined: infographics (BL 2311 and BIO 3310) and academic explanatory articles (BL 4411). Assignment instructions and analytic rubrics are provided in the supplemental materials. For the infographic assignment, students selected a topic relevant to the course and designed an infographic intended to communicate an important scientific concept to a general audience. Students completing the academic explanatory article researched an emerging ethical issue in genomic science and prepared an evidence‐based article written for a general audience. Both assignment types emphasized scientific communication, critical evaluation of evidence, and revision through formative feedback. Students whose final submissions met established criteria were eligible for publication on the StMU Research Scholars website.

**FIGURE 1:**
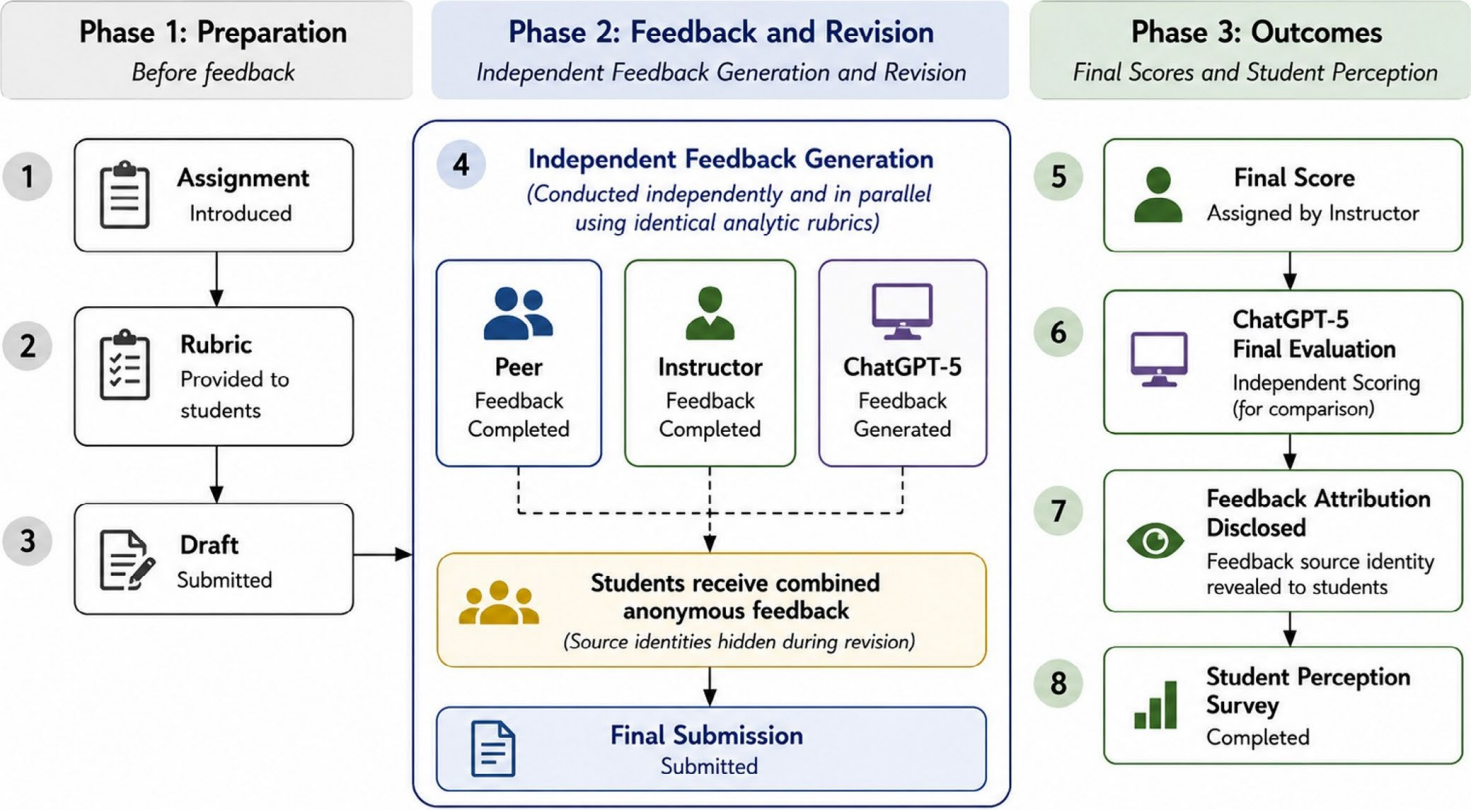
Study design and workflow. Students completed Writing‐to‐Learn assignments through a three‐phase process consisting of assignment preparation, independent formative feedback generation, revision, and post‐project evaluation. During revision, instructor, peer, and ChatGPT‐5 feedback were generated independently using the same analytic rubric. Feedback sources remained unidentified during revision and were disclosed only after instructor grading, immediately before administration of the student perception survey.

The overall study workflow is summarized in Figure 1. Both assignment types followed a scaffolded, draft‐based process, and students had access to rubrics at the onset of their assignment. Students first submitted a draft, which independently received formative feedback from three sources: the course instructor, AI (ChatGPT‐5), and peers. Peer feedback consisted of evaluations from two classmates per submission, and the two peer scores were averaged to generate a single composite score.

Reviewers evaluated student work using the same assignment‐specific analytic rubric. Students received the rubric when the assignment was introduced, throughout the drafting process, and utilized it for feedback. Peer reviewers did not receive formal training. The instructor independently applied the same rubric using their respective expertise and the assignment‐specific prompt. AI‐generated feedback was produced using ChatGPT‐5 via the OpenAI ChatGPT web interface. Each student submission was evaluated in a separate conversation with memory features disabled to ensure that no information was retained across evaluations. ChatGPT‐5 received the assignment instructions, analytic rubric, and a standardized prompt directing it to complete the rubric and provide criterion‐specific written feedback (Supplemental Data). The prompt instructed the model to: “Utilizing the uploaded assignment instructions and rubric, please provide detailed feedback on this assignment and complete the rubric. Pay careful attention to sources and citations.” AI had no access to instructor or peer scores and did not retain or learn from student submissions as memory features were turned off. Final submissions were evaluated independently by both the instructor and ChatGPT‐5 using the same analytic rubrics and prompts applied during the draft stage.

All feedback providers completed evaluations independently. The instructor completed rubric scoring without access to peer or AI‐generated evaluations, and peer reviewers completed their evaluations without access to instructor or AI feedback. This independent scoring process was maintained for both the draft (peer, instructor, and AI) and the final stages (instructor and AI) to ensure that inter‐rater agreement reflected independent assessments rather than consensus among reviewers.

### 2.3 Rubrics and Scoring

Analytic rubrics were developed by adapting previously used course assessment instruments and iterative refinement with assistance from ChatGPT‐4 (OpenAI), the LLM available during Summer 2025. Final rubric content, performance descriptors, and scoring criteria were reviewed, modified as needed, and approved by the course instructor (author) prior to implementation. Once finalized, the rubrics remained unchanged throughout the study and were applied consistently by the instructor, peers, and ChatGPT‐5 (released August 7, 2025) during student evaluation in Fall 2025.

Infographic assignments (BL 2311 and BIO 3310) were evaluated using a standardized analytic rubric consisting of five criteria: Content & Accuracy (CA), Organization & Storytelling (OS), Design & Visual Appeal (DV), Sources & Citations (SC), and Mechanics & Professionalism (MP). Each criterion was scored on a 5‐point scale, yielding a total possible score of 25. Students also received written feedback in each category.

Academic explanatory articles (BL 4411) were evaluated using a discipline‐appropriate analytic rubric adapted to include six criteria: Scientific Understanding & Accuracy (SA), Ethical Reasoning Grounded in the Catholic Intellectual Tradition and Generalized Empirical Method Framework (ER), Argumentation & Organization (AO), Sources & Citations (SC), Clarity, Mechanics, & Professionalism (MP), and Engagement & Reflection on the Broader Impact (BI). Each criterion was scored on a 5‐point scale, yielding a total possible score of 30. Because the rubrics differed in both content and scoring structure, inter‐rater reliability analyzes were conducted separately by assignment type.

Regardless of the feedback source, all students received both quantitative rubric scores and qualitative written feedback for each criterion. Instructor and AI (ChatGPT‐5) evaluations consistently included criterion‐specific narrative comments, whereas peer reviewers were encouraged, but not required, to provide written feedback in addition to rubric scores. Consequently, all students received numeric ratings from each feedback source, while the extent of narrative feedback varied among peer reviewers.

### 2.4 Statistical Analysis

All statistical analyzes were conducted in R (version 4.5.2) with R Studio utilizing the following packages: *tidyverse* for data management, *rstatix* for nonparametric and post hoc analyzes, *irr* for intraclass correlation coefficients, and *psych* for rank‐based agreement statistics (Gamer et al. 2019; Kassambara 2026; R Core Team 2024; Revelle 2026; RStudio Team 2024; Wickham 2024). Statistical significance was set at α = 0.05.

Inter‐rater agreement among ChatGPT‐5, peer, and instructor raters was assessed separately for infographics and academic explanatory article assignments using intraclass correlation coefficients (ICC). Analyses were conducted separately because assignment types utilized different analytic rubrics and represented distinct instructional contexts. To evaluate agreement among the three fixed rater types, a two‐way mixed‐effects model using absolute agreement for average measures (ICC [3, *k*]) was selected (McGraw and Wong 1996). ICC values were interpreted as poor (< 0.50), moderate (0.50–0.75), good (0.75–0.90), or excellent (> 0.90). Reliability analyses included all complete student assignments that received scores from the instructor, ChatGPT‐5, and both peer reviewers (BL 2311 = 38, BIO 3310 = 15, and BL 4411 = 23). Sample size was constrained by course enrollment and further limited to student assignments with complete evaluations from all feedback providers. Ninety‐five percent confidence intervals were calculated and reported for all ICC estimates (Mokkink et al. 2023). Inter‐rater agreement analyses were based on numeric rubric scores; written comments were collected but were not quantitatively analyzed.

### 2.5 Student Perception Survey and Analysis

Following submission of the final assignment, receipt of all formative feedback, and assignment grading, students were invited to complete an anonymous, voluntary survey assessing their perceptions of instructor, peer, and AI generated feedback. During the revision process, formative feedback from all three sources was compiled into a single document and returned without identifying the source of the individual comments, allowing students to revise their work without attribution bias. Students were informed that they would subsequently be asked to reflect on their experiences using the feedback.

One week after final assignment grades were posted, students completed a survey instrument developed for this study (Supporting Information S1). At the time of survey administration, the source of each feedback set (instructor, peer, or ChatGPT‐5) was explicitly disclosed. Consequently, survey responses reflect students' perceptions of feedback after attribution to a known source rather than blinded evaluations of feedback quality.

The survey included five‐point Likert‐scale items assessed perceived usefulness, engagement with feedback, trust in the feedback source, and effort required to apply the feedback. Survey participation was voluntary, and student respondents were not required to answer all survey items. The survey was developed specifically for this study because no validated instrument was identified that measured perceptions of instructor, peer, and AI‐generated feedback within the same WTL workflow. Differences in student ratings among the three feedback sources were evaluated using Friedman tests for repeated, nonparametric data. Analyzes included only matched, non‐missing responses for each survey item. When Friedman tests indicated significant differences, post hoc pairwise comparisons were conducted using Wilcoxon signed‐rank tests with Holm correction applied to control for multiple comparisons. Effect sizes were calculated using Kendall's *W* and interpreted as small (~0.10), moderate (~0.30), or large (> 0.50).

## 3 Results

### 3.1 Participant Characteristics and Revision Behavior

Students represented in this study included both majors and non‐majors with varied levels of academic experience. Survey response rates differed by course, with responses from 51% of students in BL 2311 Food and Nutrition (non‐majors), 59% in BIO 3310 Introduction to Bioinformatics (majors), and 85% in BL 4411 Genes, Genomes, and Genomics (majors) (Table 1). Participation in the end‐of‐study survey assessing feedback perceptions was voluntary, anonymous, and not associated with course grades. Across all courses, most students completed multiple revision cycles beyond the draft analyzed in this study, with the majority self‐reporting revising two or three times prior to final submission and/or publication on the StMU Research Scholars website (Table 1).

**TABLE 1:** Participant characteristics, course context, and assignment features.

	BL 2311: food and nutrition	BIO 3310: introduction to bioinformatics	BL 4411: genes, genomes, and genomics
	*N* = 23 respondents	*N* = 10 respondents	*N* = 23 respondents
Student population	Non‐majors	Majors	Majors
Assignment type	Infographic	Infographic	Academic Explanatory Article
Relative content complexity	Low	High	High
Course enrollment	45	17	27
Survey response rate: % (responders/enrollment)	51% (23/45)	59% (10/17)	85% (23/27)
Student academic year: %^a^ (*n*)			
1	4.3% (1)	—	—
2	52.2% (12)	40% (4)	—
3	21.7% (5)	40% (4)	8.7% (2)
4	21.7% (5)	20% (2)	82.6% (19)
4+	—	—	8.7% (2)
Self‐reported revision cycles: % (*n*)^b^			
1	—	—	9% (2)
2	13% (3)	30% (3)	52% (12)
3	74% (17)	70% (7)	22% (5)
4 or more	13% (3)	—	17% (4)
Published on StMU research scholars^c^ % (*n*)	61% (14)	90% (9)	57% (13)

^a^ Percentages reflect proportion of survey respondents within each course. All courses are taught by the same professor.

^b^ Revision cycles represent student reported revisions completed after the initial draft submission.

^c^ Publication on the StMU research scholars website was optional and limited to assignments meeting instructor‐defined quality criteria.

### 3.2 Inter‐Rater Reliability Across Assignment Modalities

Agreement among AI, instructor, and peer evaluations ranged from moderate to good across the three undergraduate biology courses (Table 2). Overall agreement for total rubric scores was good for the BL 2311 infographic assignment (ICC = 0.79, 95% CI 0.66 to 0.88) and the BL 4411 academic explanatory article (ICC = 0.80, 95% CI 0.62 to 0.91), whereas agreement for the BIO 3310 infographic assignment was moderate (ICC = 0.54, 95% CI −0.07 to 0.82).

**TABLE 2:** Inter‐rater agreement among AI, instructor, and peer feedback on first drafts of WTL assignments.

Category	ICC (3, *k*)^a^	95% CI
Course: BL 2311 food and nutrition (non‐majors, infographic)	*N* = 38 drafts^b^	
Content & accuracy (CA)	0.72	0.53 to 0.84
Organization & storytelling (OS)	0.57	0.28 to 0.75
Design & visuals (DV)	0.48	0.13 to 0.70
Sources & citations (SC)	0.74	0.57 to 0.85
Mechanics & professionalism (MP)	0.71	0.51 to 0.83
**Total overall score**	**0.79**	**0.66 to 0.88**
Course: BIO 3310 introduction to bioinformatics (majors, infographic)	*N* = 15 drafts^b^	
Content & accuracy (CA)	0.32	−0.57 to 0.74
Organization & storytelling (OS)	0.00	−1.31 to 0.62
Design & visuals (DV)	0.00	−1.31 to 0.62
Sources & citations (SC)	0.79	0.52 to 0.92
Mechanics & professionalism (MP)	0.63	0.16 to 0.86
**Total overall score**	**0.54**	**−0.07 to 0.82**
Course: BL 4411 genes, genomes, and genomics (majors, academic explanatory article)	*N* = 23 drafts^b^	
Scientific accuracy (SA)	0.65	0.33 to 0.84
Evidence & reasoning (ER)	0.76	0.54 to 0.89
Argumentation & organization (AO)	0.58	0.18 to 0.80
Sources & citations (SC)	0.36	−0.24 to 0.70
Mechanics & professionalism (MP)	0.48	−0.01 to 0.75
Broader impact (BI)	0.75	0.51 to 0.88
**Total overall score**	**0.80**	**0.62 to 0.91**

*Note:* Rubric criteria differed between infographic and academic explanatory article assignments; therefore, category‐specific ICC values should be interpreted within assignment type rather than compared directly across assignment modalities. ICC values were interpreted using commonly accepted thresholds: poor (< 0.50), moderate (0.50–0.75), good (0.75–0.90), or excellent (> 0.90). Wide confidence intervals reflect the small sample size and limited score variability within some rubric categories. The bolded values are to distinguish the total overall score. Superscript a indicates how the ICC values should be interpreted.

^a^ ICC (3, *k*): two‐way mixed, absolute agreement, average of 3 raters.

^b^ *N* represents the number of student assignments with complete ratings from all three feedback sources.

Across individual rubric criteria, agreement varied by assignment type. For the BL 2311 infographic assignment, agreement was moderate to good across most rubric categories, with lower agreement observed for Design and Visuals (ICC = 0.48). In the BIO 3310 infographic assignment, agreement was more variable, with good agreement for Sources and Citations (ICC = 0.79) but poor agreement for the Organization and Storytelling category and Design and Visuals category (both ICC = 0.00). For the BL 4411 academic explanatory article, agreement was highest for Evidence and Reasoning (ICC = 0.76) and Broader Impact (ICC = 0.75), while other categories, Sources and Citations (ICC = 0.36) and Mechanics and Professionalism (ICC = 0.48), demonstrated lower agreement (Table 2).

### 3.3 Changes in AI‐Instructor Agreement Across Revision

Agreement between AI and instructor evaluations changed differently across assignment types following revision (Table 3). For the two infographic assignments (BL 2311 and BIO 3310), overall agreement decreased modestly from the draft to the final submission (BL 2311: ICC = 0.66 to 0.62; BL 3310: ICC = 0.54 to 0.29). Most rubric categories demonstrated stable or reduced agreement following revision, although Mechanics and Professionalism increased slightly in BL 2311.

**TABLE 3:** Changes in agreement between AI and instructor evaluations following student revision.

Category	Draft AI vs. instructor	Final AI vs. instructor	Δ ICC^c^
	ICC (3, *k*)^a^	ICC (3, *k*)^a^	
Course: BL 2311 food and nutrition (non‐majors, infographic)	*N* = 38^b^		
Content & accuracy (CA)	0.62	0.11	−0.51
Organization & storytelling (OS)	0.51	0.28	−0.23
Design & visuals (DV)	0.41	0.25	−0.16
Sources & citations (SC)	0.72	0.56	−0.16
Mechanics & professionalism (MP)	0.54	0.62	**+0.08**
Total Overall score	0.66	0.62	−0.04
Course: BIO 3310 introduction to bioinformatics (majors, infographic)	*N* = 15^b^		
Content & accuracy (CA)	0.00	0.00	0.00
Organization & storytelling (OS)	0.00	0.17	**+0.17**
Design & visuals (DV)	0.00	0.17	**+0.17**
Sources & citations (SC)	0.66	0.66	0.00
Mechanics & professionalism (MP)	0.21	0.00	−0.21
Total overall score	0.54	0.29	−0.25
Course: BL 4411 genes, genomes, and genomics (majors, academic explanatory article)	*N* = 23^b^		
Scientific accuracy (SA)	0.39	0.60	**+0.21**
Evidence & reasoning (ER)	0.52	0.73	**+0.21**
Argumentation & organization (AO)	0.31	0.73	**+0.42**
Sources & citations (SC)	0.16	0.25	**+0.09**
Mechanics & professionalism (MP)	0.24	0.00	−0.24
Broader impact (BI)	0.50	0.77	**+0.27**
Total overall score	0.72	0.80	**+0.08**

*Note:* Rubric categories differ by assignment type and are not directly comparable across assignment types. ICC values were interpreted as poor (< 0.50), moderate (0.50–0.75), good (0.75–0.90), or excellent (> 0.90). Bolded values are the positive Delta ICC values which show increased agreement. This is more specifically identified in the c superscript now.

^a^ ICC (3, *k*): two‐way mixed, absolute agreement, average of 2 raters.

^b^ *N* represents the number of student assignments with complete ratings from both feedback sources.

^c^ Δ ICC: Positive values indicate increased agreement following revision; negative values indicate decreased agreement.

In contrast, agreement between AI and instructor evaluations improved for the academic explanatory article assignment (BL 4411), with the overall ICC increasing from 0.72 to 0.80. Increases were observed across several rubric dimensions, including Scientific Accuracy, Evidence and Reasoning, Argumentation and Organization, and Broader Impact; in contrast, Mechanics and Professionalism demonstrated reduced agreement (Table 3).

### 3.4 Student Perceptions of Feedback Sources

Student perceptions of feedback differed across feedback sources for the infographic assignments but were more similar for the academic explanatory article assignment (Table 4). For students completing infographics, significant differences were observed for all survey constructs except the perceived effort required to implement feedback (*p* = 0.345). Post hoc Wilcoxon signed‐rank tests with Holm correction showed that instructor feedback consistently received higher ratings than peer feedback across usefulness items such as identifying weaknesses, improving writing quality, providing actionable suggestions, identifying scientific inaccuracies, and overall usefulness. Peer and AI‐generated feedback were rated similarly for most measures, although peer feedback was trusted and incorporated more frequently than AI feedback. Trust in the feedback source demonstrated the largest observed effect size (*W* = 0.44).

**TABLE 4:** Student perceptions of instructor, peer, and AI feedback across assignment types.

Feedback dimension	Survey item	*χ* ^*2*^ ^a^	*p* ^c^	Kendall's *W* ^a^	Significant pairwise differences (Holm‐adjusted)^b^
Infographics (food and nutrition & introduction to bioinformatics) *N* = 34 assignments					
Usefulness	Identified weaknesses	25.30	**< 0.001**	0.372	Instructor > Peer = AI
	Specific & actionable	19.80	**< 0.001**	0.291	Instructor > Peer = AI
	Improved organization/clarity	23.30	**< 0.001**	0.343	Instructor > Peer = AI
	Improved scientific accuracy	16.30	**< 0.001**	0.240	Instructor > Peer = AI
Engagement	Incorporated feedback	18.70	**< 0.001**	0.275	Instructor > Peer > AI
	Understood rationale	15.80	**< 0.001**	0.232	Instructor > Peer = AI
Trust	Trusted the source	29.90	**< 0.001**	0.440	Instructor > Peer > AI
Effort	Effort to apply feedback	2.13	0.345	0.031	No significant differences
Academic explanatory article (genes, genome, and genomics) *N* = 19 assignments					
Usefulness	Identified weaknesses	9.88	**0.007**	0.260	Instructor > Peer = AI
	Specific & actionable	9.37	**0.009**	0.246	Instructor > Peer = AI
	Improved organization/clarity	5.90	0.052	0.155	No Significant Differences
	Improved scientific accuracy	8.00	**0.018**	0.211	Instructor > Peer = AI
Engagement	Incorporated feedback	7.60	**0.022**	0.200	No significant differences
	Understood rationale	6.55	**0.038**	0.172	No significant differences
Trust	Trusted the source	13.50	**0.001**	0.356	Instructor > Peer = AI
Effort	Effort to apply feedback	2.80	0.247	0.074	No significant differences

*Note:* Bold *p* values indicate statistical significance at *p* < 0.05.

^a^ *χ* ^2^ values are from Friedman tests comparing Peer, Instructor, and AI feedback. Effect sizes are reported as Kendall’s W. Degrees of freedom for all tests = 2.

^b^ Directional summaries are based on Holm‐adjusted Wilcoxon signed‐rank tests following significant Friedman tests.

^c^ Statistical significance was set at α = 0.05.

Among students completing academic explanatory articles, fewer differences were observed across feedback sources, and effect sizes were generally smaller (*W* = 0.15–0.36). Significant differences were limited primarily to identifying weaknesses, specificity, scientific accuracy, and trust, with instructor feedback rated higher than both peer and AI feedback. No significant differences were observed for organization and clarity, engagement metrics, or the perceived effort required to apply feedback (Table 4). Across both assignment types, students reported comparable levels of effort when incorporating instructor, peer, and AI feedback into revisions.

### 3.5 Student‐Reported Reasons for Rejecting AI Feedback and Perceived Learning Gains

Students identified several reasons for choosing not to incorporate AI‐generated feedback into their assignment revisions (Table 5) (respondents were allowed to select multiple reasons). The most frequently reported concern was that AI feedback conflicted with feedback from another source (55%), followed by concerns that the feedback was inaccurate or unclear (37%). Of the respondents, 29% indicated that AI feedback did not align with the assignment rubric.

**TABLE 5:** Student‐reported reasons for rejecting AI‐generated feedback during revision.

Reason for rejecting AI feedback	% (*n*)
Conflicted with other feedback	55% (27)
Inaccurate content	37% (18)
Unclear	37% (18)
Didn't match rubric	29% (14)
Conflicted with course materials	18% (9)
Time	16% (8)

*Note:* *N* = 49 respondents who reported rejecting at least one AI suggestion in the survey; multiple responses were permitted.

Despite these concerns, students generally reported positive outcomes with using AI‐generated feedback (Table 6). Thirty‐nine percent of respondents indicated that AI feedback improved the quality of their final product, while an additional 41% responded that it may have improved their work. Most students also reported perceived improvements in their ability to communicate scientific concepts (80%) and their understanding of biological content (84%) following the completion of the project.

**TABLE 6:** Student perceptions of learning outcomes and contributions of AI feedback.

	Yes, % (*n*)	Maybe, % (*n*)	No, % (*n*)
“My ability to communicate science improved with this project”	80% (39)	16% (8)	4% (2)
“My understanding of biology content improved”	84% (41)	16% (8)	—
“The final product (infographic or article) quality improved because of AI feedback”	39% (19)	41% (20)	20% (10)
“AI feedback improved accuracy of labels, units, and figure captions”	35% (17)	29% (14)	37% (18)
“AI feedback improved visual hierarchy and readability”	47% (23)	39% (16)	20% (10)
“AI feedback reduced misleading or exaggerated claims”	35% (17)	37% (18)	29% (14)

*Note:* Percentages are based upon responses from students who completed the end‐of‐project survey (*N* = 49 respondents).

## 4 Discussion

This study examined the alignment of generative AI feedback, instructor, and peer feedback across two WTL assignment modalities (academic explanatory articles and infographics) implemented in undergraduate biology courses. The present findings should be interpreted as evidence regarding one implementation of AI‐assisted formative feedback and not definitive evidence regarding the capabilities of any individual large language model. Overall, agreement between AI and instructor evaluations ranged from moderate to good and varied according to assignment type, while students consistently rated instructor feedback as the most useful and trustworthy source. Although students revised their work using feedback without knowing its source, perceptions were collected only after feedback sources were disclosed. Consequently, the survey findings reflect students' evaluations of identified feedback sources under authentic classroom conditions, where judgments of usefulness and trust are naturally influenced by instructor authority and source attribution. Importantly, AI was used within a structured instructional workflow that incorporated assignment‐specific prompts and analytic rubrics rather than as an unrestricted generative tool. Collectively, these findings support the use of large language models as formative instructional supports that complement, rather than replace, instructor expertise in undergraduate STEM education (Liew and Tan 2025; Er et al. 2025; Tossell et al., n.d.; Holmes et al. 2019; Pan 2025; Henderson et al. 2026).

### 4.1 Assignment Modality and Course Complexity Matter

Agreement between AI and instructor feedback was consistently higher for the academic explanatory articles and weaker for the infographics. This pattern suggests that LLMs may demonstrate greater alignment with instructor evaluations when assessing text‐based scientific communication, where rubric criteria emphasize scientific reasoning, organization, and written argumentation. Similar observations have been reported in previous studies demonstrating stronger performance of LLMs on writing‐intensive assessments than on tasks requiring evaluation of multimodal or visual communication (Yore et al. 2004; Mercer‐Mapstone and Kuchel 2016; Drinkwater Gregg et al. 2025).

In contrast, agreement was lower and more variable for infographics, particularly in BIO 3310 (a majors bioinformatics course vs. nonmajors nutrition course) (Table 2). Unlike written assignments, infographic evaluation required interpretation of visual design, representational choices, and the integration of scientific content with graphical communication. Prior research has shown that multimodal and visual representations impose additional cognitive and interpretive demands that can complicate both learning and assessment (Hand and Prain 2002; McDermott and Hand 2016). Notably, the use of a consistent rubric and assignment format did not guarantee stable agreement across instructional contexts, underscoring that assignment modality may influence the reliability of AI‐assisted formative feedback. Although ChatGPT‐5 demonstrated useful agreement across both assignment types, the strongest alignment with instructor evaluations was observed for text‐based scientific writing.

### 4.2 Student Experience and Scientific Communication

Prior studies in STEM education suggest that informal, representational writing tasks, such as infographics, can promote conceptual understanding and scientific communication skills, particularly among novice learners (Hand and Prain 2002; Reynolds et al. 2012). Consistent with this literature, most students in the present study reported perceived improvements in both their ability to communicate science and their understanding of biological content following the completion of the assignments (Table 6). These findings suggest that AI‐supported formative feedback may be incorporated into WTL activities without diminishing students' perceived learning benefits.

Greater agreement between AI and instructor evaluations was observed for the upper‐level academic explanatory article assignment than for the introductory infographic assignment. Although this difference may reflect increased student experience with disciplinary writing conventions, it may also be influenced by differences in assignment modality, rubric structure, or course context. Notably, agreement between AI and instructor evaluations remained lower for Mechanics and Professionalism on final academic explanatory articles, suggesting that instructor judgment may have reflected a more nuanced application of rubric criteria related to scientific writing (Table 3). This finding is consistent with previous work emphasizing the importance of expert feedback for evaluating scientific precision, conceptual integration, and rhetorical effectiveness in advanced science communication (Brownell et al. 2013a; Yore et al. 2004).

### 4.3 Pedagogical Implications for AI‐Assisted Feedback in STEM Courses

Taken together, these findings support the use of AI tools as deliberate, stage‐specific instructional scaffolds that complement instructor expertise. Students consistently perceived instructor feedback as more useful and trustworthy than both peer and AI‐generated feedback (Table 4), reinforcing the continued importance of instructor expertise in providing disciplinary guidance and evaluating complex scientific reasoning (Hattie and Timperley 2007; Cho and MacArthur 2010; Henderson et al. 2026). At the same time, students reported comparable levels of effort required to incorporate feedback across all sources, suggesting that AI‐generated feedback does not impose additional cognitive burden. Moreover, AI feedback was frequently perceived as similar to peer feedback, positioning it as a potentially valuable addition to multi‐source formative feedback environments.

Rather than framing AI feedback in terms of equivalence to instructor feedback, a more meaningful question is when and how generative AI formative feedback can best support student learning in the constructs of existing educational practices. Responsible implementation also requires transparency regarding the role of AI in the feedback process, continued instructor oversight, and opportunities for students to critically evaluate AI‐generated suggestions and not just accept them uncritically. Such practices promote ethical, inclusive, and student‐centered use of AI by preserving instructor accountability while encouraging students to develop feedback literacy and disciplinary judgment.

Within WTL assignments, AI feedback may help students identify baseline weaknesses, clarify assignment expectations, and engage more effectively in early revision cycles, consistent with formative assessment theory (Hattie and Timperley 2007). In this way, AI‐supported feedback has the potential to reduce instructor burden while preserving instructional time for higher‐level conceptual reasoning, disciplinary nuance, scientific communication, and evaluation. Instead of focusing exclusively on improvements in AI capability, educators should consider how LLMs are incorporated into existing feedback practices in ways that promote transparency, instructor oversight, and inclusive, student‐centered formative feedback. The present findings suggest that structured implementation using analytic rubrics, assignment‐specific prompts, and instructor oversight can support the effective integration of AI‐generated formative feedback into undergraduate STEM courses.

### 4.4 Limitations and Future Directions

This study has several limitations that should be considered when interpreting the findings. The study was conducted at a single institution using three undergraduate biology courses with modest enrollment, which may limit the generalizability of the results. Additionally, assignment type, course context, and student experience differed across courses and could not be evaluated independently, limiting the ability to attribute differences in agreement or student perceptions to any single factor.

Students received feedback from AI, peers, and instructors simultaneously and integrated feedback without source attribution during revision. Feedback sources were disclosed before administration of the post‐project survey. Consequently, survey responses reflect students' perceptions of identified feedback sources rather than blinded evaluations of feedback quality and likely incorporate perceptions of source credibility in addition to judgments of feedback usefulness.

Future studies should examine whether student perceptions differ when feedback sources remain blinded, compare multiple LLMs within the same instructional workflow, and investigate how students decide which AI‐generated suggestions to incorporate during revisions. Larger multi‐institutional studies involving a broader range of STEM disciplines and assignment types would further clarify the contexts in which AI‐assisted formative feedback is most effective. Additional future studies should also explore students' open‐ended reflections on AI‐generated feedback to better understand how learners evaluate, accept, or reject AI suggestions during the revision process. As AI systems continue to evolve, continued empirical work is needed to identify instructional contexts in which AI feedback can responsibly enhance learning while maintaining the central role of expert human judgment (Holmes et al. 2019; Pan 2025; Henderson et al. 2026).

### 4.5 Concluding Perspective

Within the courses and assignments examined in this study, agreement between AI‐generated feedback and instructor evaluations varied according to assignment type and instructional context. When integrated strategically as a formative scaffold within Writing‐to‐Learn frameworks, AI‐generated feedback can support student revision without increasing perceived effort and function comparably to peer feedback. However, instructor expertise remained essential for evaluating conceptual depth, disciplinary reasoning, scientific communication, and rhetorical nuance. Large language models, like ChatGPT‐5, should not be viewed as surrogates for instructor feedback but as structured formative tools whose value depends on thoughtful integration into existing instructional practice. When implemented with clear analytic rubrics, assignment‐specific prompts, and instructor oversight, they have the potential to strengthen formative feedback practices while preserving the central role of expert human judgment in undergraduate STEM education.

## Author Contributions

**Lori Boies:** conceptualization (lead), data curation (lead), formal analysis (lead), investigation (lead), methodology (lead).

## Funding

The author has nothing to report.

## Ethics Statement

This study design was considered exempt (study is program evaluation and is not regulated research under CFR Section 46.102) by the St. Mary's University Institutional Review Board, and all participating students completed an informed consent form.

## Conflicts of Interest

The author declares no conflicts of interest.

## Supporting information


**Data S1:** ece374365‐sup‐0001‐Supinfo.pdf.

## Data Availability

De‐identified data supporting the findings of this study, including rubric scores and survey responses, are available from the corresponding author upon reasonable request. Student‐generated assignments are not publicly shared to protect participant confidentiality. Assignment instructions/materials, including analytic rubrics, are provided in the supplemental material.
