# Supplementary material for: Evaluating Artificial Intelligence, Peer, and Instructor Feedback Across Scientific Communication Assignments in Undergraduate Biology
Source: Ecol Evol. 2026 Sep 20;16(9):e74365. doi: 10.1002/ece3.74365 (PMC13590280; doi:10.1002/ece3.74365)
Supplement: Supplementary file 1 [file ECE3-16-e74365-s001.pdf]

BL / BIO 4411 – Genes, Genomes, and Genomics

Course Project – Ethical Concerns in Genomics

As the field of genomics and the application of biotechnology greatly expands, society finds itself on the precipice of a brave new world in which not only scientists but also policymakers will be responsible for deciding the limits of what is ethical. Students in BIO/BL 4411: Genes, Genomes, and Genomics are asked to not only learn the scientific underpinnings of genomics, but also to interact with the material in the broader context of how it can be applied to our understanding of inheritance of traits, personalized medicine, genetically modified organisms, and many other facets. Integral to these concepts is also the ethical considerations.

Upon reading the following quote from the textbook, "Human Heredity: Principles and Issues." by Michael R. Cummings, think about a pressing ethical issue that you think would be important to address.

> ***What Choices Do We Make in the Era of Genomics and Biotechnology?*** [1]
>
> *"In the span of about 40 years, we have learned how to predict the sex of unborn children, diagnose many genetic disorders prenatally, and manufacture human gene products to treat genetic diseases. We are now at a transition point where we are not only learning more about human genetics, but we are starting to apply genetic knowledge in ways that were unforeseen just a few years ago. These applications are colliding with social standards, public policy, and laws, forcing us to rethink what is acceptable and unacceptable in our personal and public lives.*
>
> *Should we buy and eat food that comes from genetically modified plants and animals? Is milk from cloned cows safe to drink? Should we test ourselves or our children for genetic diseases even if no treatment is available? Is medicine produced from genetically modified animals safe? Should we vaccinate our children with edible vaccines produced from genetically altered plants? We are faced with an increasing number of seemingly bewildering choices. Sorting through the rhetoric and hype to find the facts that allow us to make intelligent and informed choices is a problem in modern life. Beyond these immediate personal choices is the fact that the development of biotechnology is raising new ethical questions that we must face and answer in the near future.*
>
> *We can make informed personal decisions and formulate relevant laws and public policy only if we have a working knowledge of the principles of genetics as they apply to humans and understand how genetics is used in biotechnology. As a student of human genetics, you have elected to become involved in the search for answers to these important questions."*

As a scholar at a Catholic, Marianist University, the ethical issue that is of interest to you will be investigated through the lens of Catholic Intellectual Tradition (CIT) as influenced by Benard Lonergan. Lonergan recognized that there are many different types of knowledge to include (but not limited to): common sense, interpersonal relations, scholarship, self-knowledge, philosophy, etc. Through exploring Lonergan's framework of intentional consciousness, the topic will be explored through the four transcendental imperatives:

- Being Attentive; *in* ***Experience***
- Being Intelligent; *in* ***Understanding***
- Being Reasonable; *in* ***Judgement***
- Being Responsible; *in* ***Deciding***

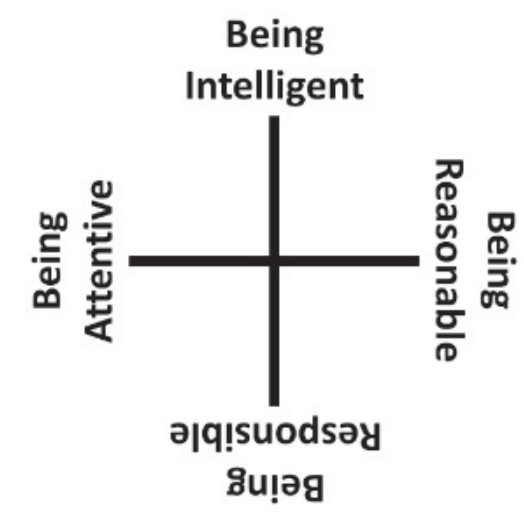

Throughout this semester long project, you will be utilizing Lonergan's "generalized empirical method," (GEM). Please reference the recorded lecture for more information about the GEM and this project. For more information about GEM and Lonergan, please visit the following sites:

- https://iep.utm.edu/lonergan/
- https://equivalentexchange.blog/2015/12/09/the-four-transcendental-imperatives-of-bernard-lonergan/

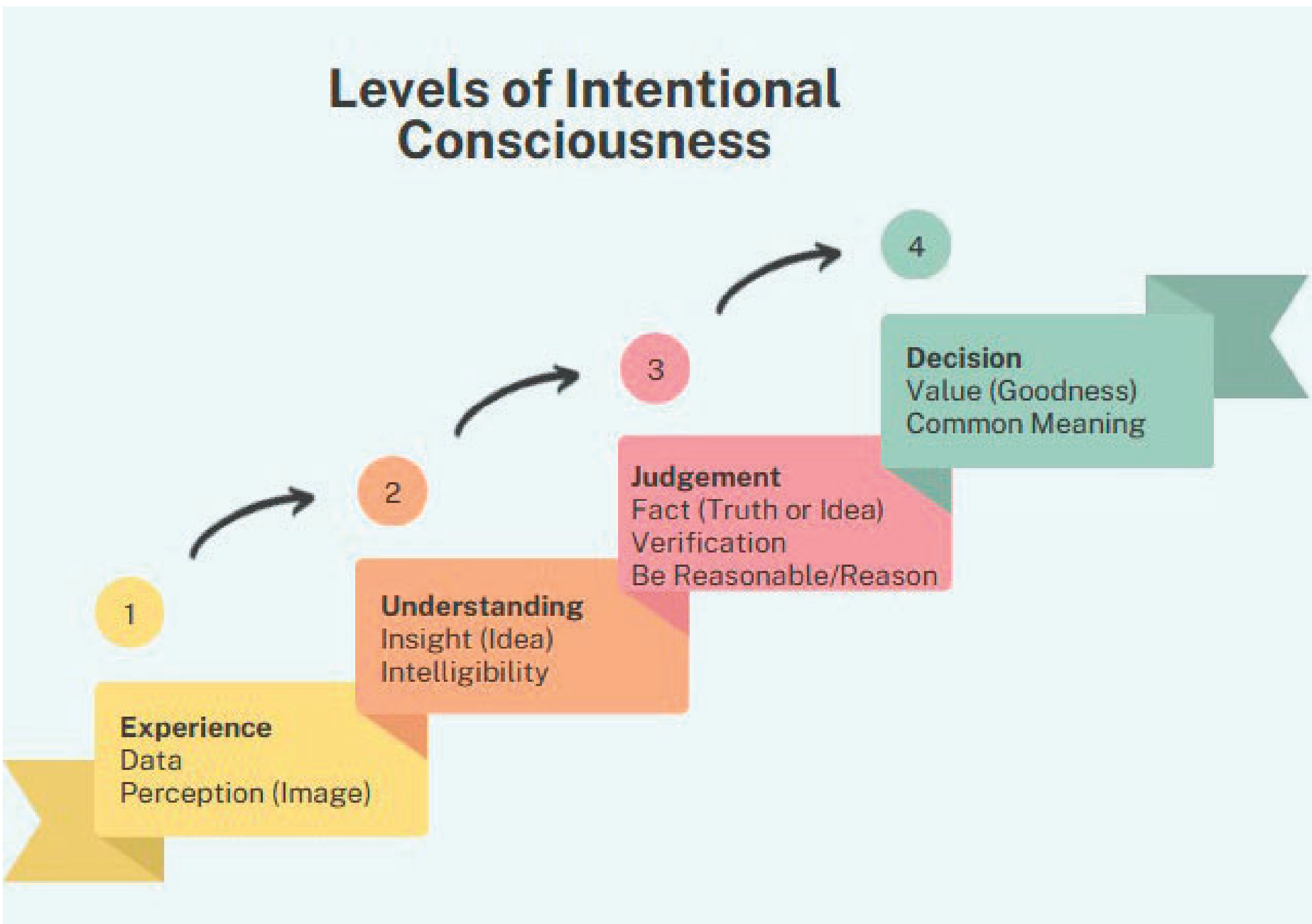

GEM Level	*Assignment*	*Components*	*Audience*	*Due Date*
**Experience**	Research Project Proposal	• What is the topic you would like to explore?<br>• What biases/beliefs are you bringing into the project on this topic?<br>• What is the relevant evidence? (Cursory/initial literature review)	Your Professor	September 8
**Understanding**	Explanation of Topic to Peers – Discussion Board	• Create an easily interpretable summary of the two (or more) points of view you are exploring in your ethical concern topic. (See handout for more information).<br>• Provide peers with a summary paragraph of the points outlined.<br>• Please provide citations.	Your Professor and Peers	September 15
**Judgement**	Meaningful Dialogue on Topic Discussion Board	• Students are asked to pick two topics that are of interest and provide their insight on the topic.<br>• This level of judgement should take the insights provided and create judgements on facts and values. (See handout)	Your Professor and Peers	September 29
**Decision**	Final Research Project	• 1st Draft (10-06)<br>○ Peer Review two drafts (10-13)<br>• Final Draft (10-27)<br>• Final Publication on the StMU Research Scholars Site (11-3)	Your Professor, Peers, and the General Public	October 6<br>October 13<br>October 27<br>November 3

*Please see corresponding assignment handout for Understanding and Judgement.

Knowing is a three-fold composite of experiencing, understanding, and judging.

Knowledge is a three-fold composite of relevant data, intelligibility, and verified fact that is "isomorphic" with the structure of consciousness.

You are required to thoroughly research and write an Academic Explanatory article to be published on the StMU Research Scholars Page: https://stmuscholars.org/

- Students are to pick their own topics. Dr. Boies will make sure that there is minimal overlap between topics. Students who submit their topics first will be given priority over similar topics submitted later.
    - Students are encouraged to submit early to ensure that they can write on the topic that they are interested!
    - If you are having problems picking a topic, please set up an office hour appointment and I happy to speak with you!
- Minimum of 2000 words
- Citations: The Chicago style of formatting is preferred for the StMU Research Scholars Site. The following will also be accepted for this assignment: APA formatting or the numbered IEEE style
- A ***minimum*** of 7 scholarly sources (you can have more!!)
- Students are required to meet with the RSC (Rattler Success Center) tutors at least once and provide documentation that you did meet with a writing tutor.
- The essence of the Academic Explanatory article is its focus on a question or problem of scholarship, where other scholars would acknowledge that that question or problem is worthy of research energy. Students need to define their research by focusing on the contributions that other scholars have already invested in those questions or problems. Students need to master the arguments used by those scholars, analyze those scholars' use of evidence and argumentation, and then make up their own minds about the answer to their question or problem, which would become their thesis for their own answer to that question or problem of scholarship.
    - I encourage to students to make appointments with the research librarians! (Diane Duesterhoff and Anacely Murphy)
- Your goal is to then design an argument to explain the question or problem to your readers, enter into a conversation with those other scholars, and persuade your readers of your understanding of the case.
- **What you DO NOT want to do is simply give your uninformed opinion about something, like a reflection paper.**
- **As you are publishing this on the StMU Research Scholars site (a public site), you can place this publication on your resume/CV. This should be a project and product that you are proud of!**

Important Dates:

- September 8: Research Project Proposal Due
- October 6: Draft of research paper due
  - Students will be asked to peer-edit; due October 13
- October 27: Research paper due
- November 3: Publication cut-off date for StMU Research Scholars Nomination
- November 18: Nominations for Awards Posted
- November 25 – 26: StMU Research Scholars Voting Days
- **December 5: Award Ceremony (6:30 pm in UC-Room A)**
  - Students are required to attend the award ceremony if they have been nominated!

Miscellaneous:
Students are encouraged to discuss any questions, etc. about the project with Dr. Boies **throughout the semester** as they are working on paper. Questions submitted to Dr. Boies at the "last-minute" before a part of the project is due *are not guaranteed to be answered in-time for the student to make appropriate changes before the assignment is due*.
Office Phone: 210-436-3521
Email: lboies@stmarytx.edu
Book Office Hour Appointments: www.calendly.com/lboies
Any problems with Canvas that cannot be resolved using the help menu should be reported to the Help Desk of Technology Services (email: helpdesk@stmarytx.edu; phone: 210-431-4357) so that these issues can be addressed and resolved. Unfortunately, I am not IT and cannot help you with these issues! If you email me, I will tell you the following:
"Please contact the help desk to address your issues."

Unforeseen technology issues are a reason that students should not wait until the "last-minute" to submit their assignments. 😊

Good luck and have fun!

Citations:

1. Cummings, M. R. (2015). *Human heredity: Principles and issues*. Cengage Learning.

Genes, Genomes, and Genomics
Draft Feedback Rubric

Article Title:

	5	4	3	2	1	Points Awarded
**Scientific Understanding and Accuracy**<br>• Does the article demonstrate clear comprehension of genomic science relevant to the ethical issue?<br>• Are claims accurate, evidence-based, and contextualized using scholarly sources?	Scientific explanations are thorough, accurate, and reflect deep understanding of genomic principles.	Minor gaps or simplifications, but mostly accurate.	Some misunderstanding or oversimplification.	Several inaccuracies weaken the argument.	Major factual errors; lacks scientific grounding.	
**Written Feedback for Scientific Understanding and Accuracy:**						
**Ethical Reasoning through Catholic Intellectual Tradition (CIT) and GEM Framework**<br>• Does the student engage ethically with the topic through Lonergan's four levels (Experience, Understanding, Judgment, Decision)?<br>• Are moral and social implications thoughtfully explored?	Demonstrates insight across all four GEM levels; integrates CIT perspectives clearly and responsibly.	Engages ethically and intellectually with strong CIT application; minor gaps in depth.	Some ethical analysis, but CIT/GEM integration is partial or uneven.	Limited ethical reflection or lacks connection to GEM.	No substantive ethical reasoning.	
**Written Feedback for Ethical Reasoning:**						
	**5**	**4**	**3**	**2**	**1**	**Points Awarded**

**Argumentation & Organization**<br>• Is there a clear thesis framed as a scholarly question or problem?<br>• Does the paper flow logically from evidence to conclusion?	Thesis is precise and original; argumentation is logical, well-structured, and persuasive.	Clear organization; minor issues in cohesion or transition.	Reasonable structure but uneven development of ideas.	Disorganized or repetitive; thesis unclear.	No discernible argument or structure.	
**Written Feedback for Argumentation & Organization:**						
**Sources & Citations**<br>• Are in-text citations consistently and accurately used to support claims?<br>• Are sources authoritative, current, and relevant to genomics and ethics?<br>• Are citation styles consistent and complete (Chicago, APA, or IEEE)?	Extensive and effective use of scholarly sources; all claims supported with accurate in-text citations; reference list complete and consistent.	Strong source integration with minor citation or formatting inconsistencies.	Adequate number of sources, but integration is uneven or several claims lack citations.	Frequent missing or incorrect in-text citations; weak connection between claims and sources.	Minimal or absent citations; no evidence of proper attribution.	
**Written Feedback for Sources and Citations:**						
**Clarity, Mechanics & Professionalism**<br>• Is the draft polished, coherent, and appropriate for public publication (StMU Scholars)?<br>• Is grammar, syntax, and tone professional?	Clear, concise, and error-free; academic tone sustained.	Minor errors that do not impede understanding.	Some grammatical or stylistic issues.	Frequent errors that distract from meaning.	Unpolished and unclear.	
**Written Feedback for Mechanics and Professionalism:**						

	5	4	3	2	1	Points Awarded
**Engagement & Reflection on Broader Impact**<br>• Does the article connect genomics and ethics to broader human, societal, or Marianist concerns?	Insightful connection between scientific content, ethics, and societal implications; demonstrates awareness of global or communal impact.	Clear ethical engagement; connects science to broader human context.	Some attempt to address broader implications but lacks depth.	Minimal reflection on ethics beyond surface-level commentary.	No broader reflection evident.	
**Written Feedback for Engagement & Reflection on Broader Impact:**						
					**Total Points Awarded**	
**General Comments and Feedback:**						

BIO / BL 3310 – Introduction to Bioinformatics
Infographic Project Instructions

Step-by-step guide for BIO/BL 3310 – Introduction to Bioinformatics

In BIO/BL 3310 for your research project, you will create an infographic and write a research outline instead of the traditional original research paper to communicate your research findings. While the traditional original research format is suitable for many different fields and types of studies, this is not the only way to present research and is not always the preferred format for a variety of reasons (e.g., cost and/or obscurity of journal, timing issues with sensitive data, excessive jargon that deters public connection and consumption, audience, etc.). In this infographic project, you are compiling secondary data findings into an analysis to be visually presented as a combination of numbers, text, and images. This infographic will convey research with a clear emphasis on purpose, design, readability, and types of visual representation (e.g., picture, graph, chart, etc.). Your goal is to produce an infographic that: 1) allows a reader to derive the scholarly meaning from it; 2) is sound with meaning and integrity; and 3) is aesthetically appealing (Lankow, Ritchie, & Crooks, 2012).

Additionally, it is important to be able to relay important information quickly in an easy-to-understand format. For this project, you will convey, through an infographic, the importance of the growing bioinformatics field, potential career opportunities in bioinformatics, or another topic that conveys "what is" bioinformatics.

There are three key objectives to this project:

A. Translating key empirical finding into a visually communicated message that includes graphs or charts, a narrative, descriptive text, icons and/or images.
B. Developing digital proficiency in infographics in both content creation and visual understanding (e.g., use of color and white space, text boxes, font sizes, placement of text, universal design, etc.).
C. Demonstrating an ability to use technology as a form of research storytelling.

Goals assessment:

1. Evaluation of the ability to use library refereed sources to collect public health data and organize, evaluate, and select data that is appropriate for the infographic.
2. Evaluation of the design, coherence, and readability of the infographic.
3. Encouragement to share the infographic with other publics and individuals outside the university through StMU Research Scholars.

Your final infographic will consist of text annotation and visualizations that convey your selected research topic framed by scholarly data. You will create a one-page infographic that **includes five key points** with at least **one numeric data point**. You may use Canva (www.canva.com - there are free educational accounts!) or other similar website to create your infographic using a presentation or infographic template. If you want to make an interactive infographic, you can Genially also has free templates that you can use (https://genially.com/create/infographics/). Well-executed interactive infographics may earn bonus points on the assignment (not guaranteed, this is left to instructor discretion).
The following is a guide to the development and successful completion of your infographic. Space and/or text boxes are provided for you to fill in your work as it develops.

- Infographics need to be 1600 – 1700 pixels wide to display at their fullest.
    - The "resize" setting is in the upper left corner of Canva.
    - For formatting, portrait orientation is preferred

A. Title of Infographic (*less than 12 words, descriptive, interesting to readers, accurate to content)*

B. The story. What is the story you are telling? The story should dictate the design and reflect the research purpose (e.g., descriptive, exploratory, explanatory, evaluation). Use the following questions to get at your data viz story: *What story am I telling? What do I already know about it and what will I need to research? Where will I get that information? Why does this story need to be told? Who will be my audience? What does my audience need to know and how will they want to learn it? What do you want your audience to **do** with this information?*

C. Central data points/annotations.

What is the order of your key points? *(think narrative arc)*

What is the summarizing/concluding statement (the “so what” factor)?

What is the central hook or take-away? “Hero” statistic? Where will you visually locate this hook (e.g., center, at the end)?

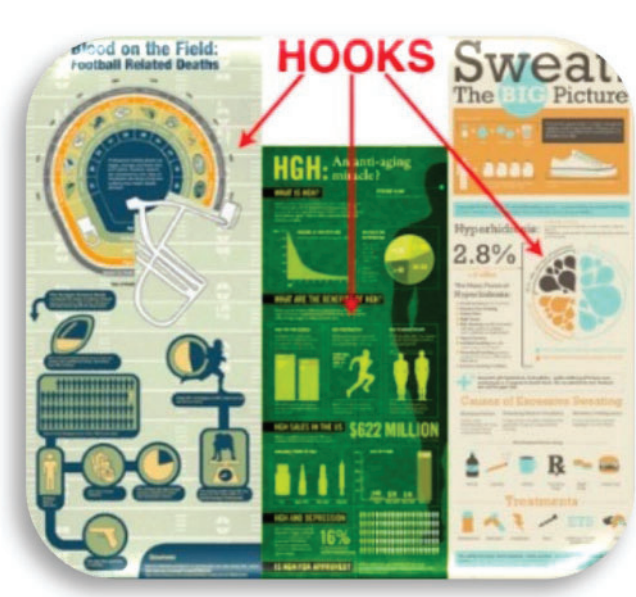

D. Additional scholarly and non-scholarly references. Use APA the numbered IEEE style format.

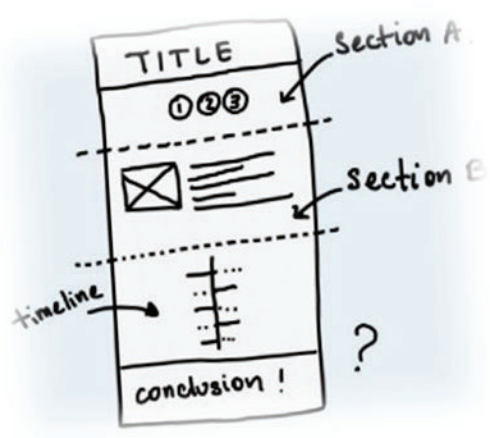

- **Infographic Sketch also referred to as "a wireframe" or a visual representation that demonstrates the relationship of elements on a page. Select draft layout, text, and visuals.**
- 9 x 11" standard size (saved as jpeg or PDF)—vertical or horizontal
- *Before you sketch, consider type of visual representation (process, flow, comparison, timeline, etc.) that would make the most sense for conveying the information needed.*

Example of an infographic; the Texas Medical Association has some great examples! www.texmed.org

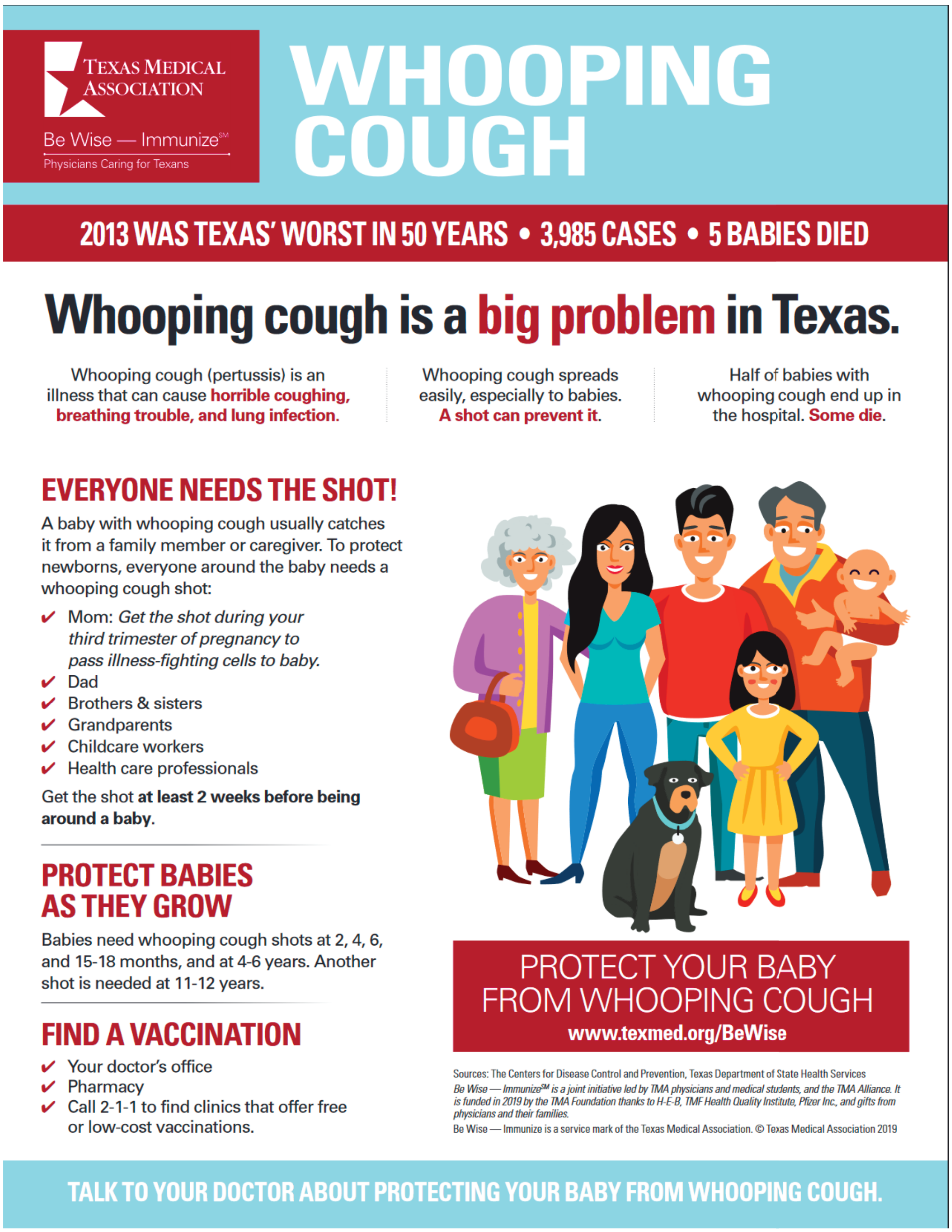

Here are two resources for you to consider as you create a layout and choose charts, graphics, and visuals.

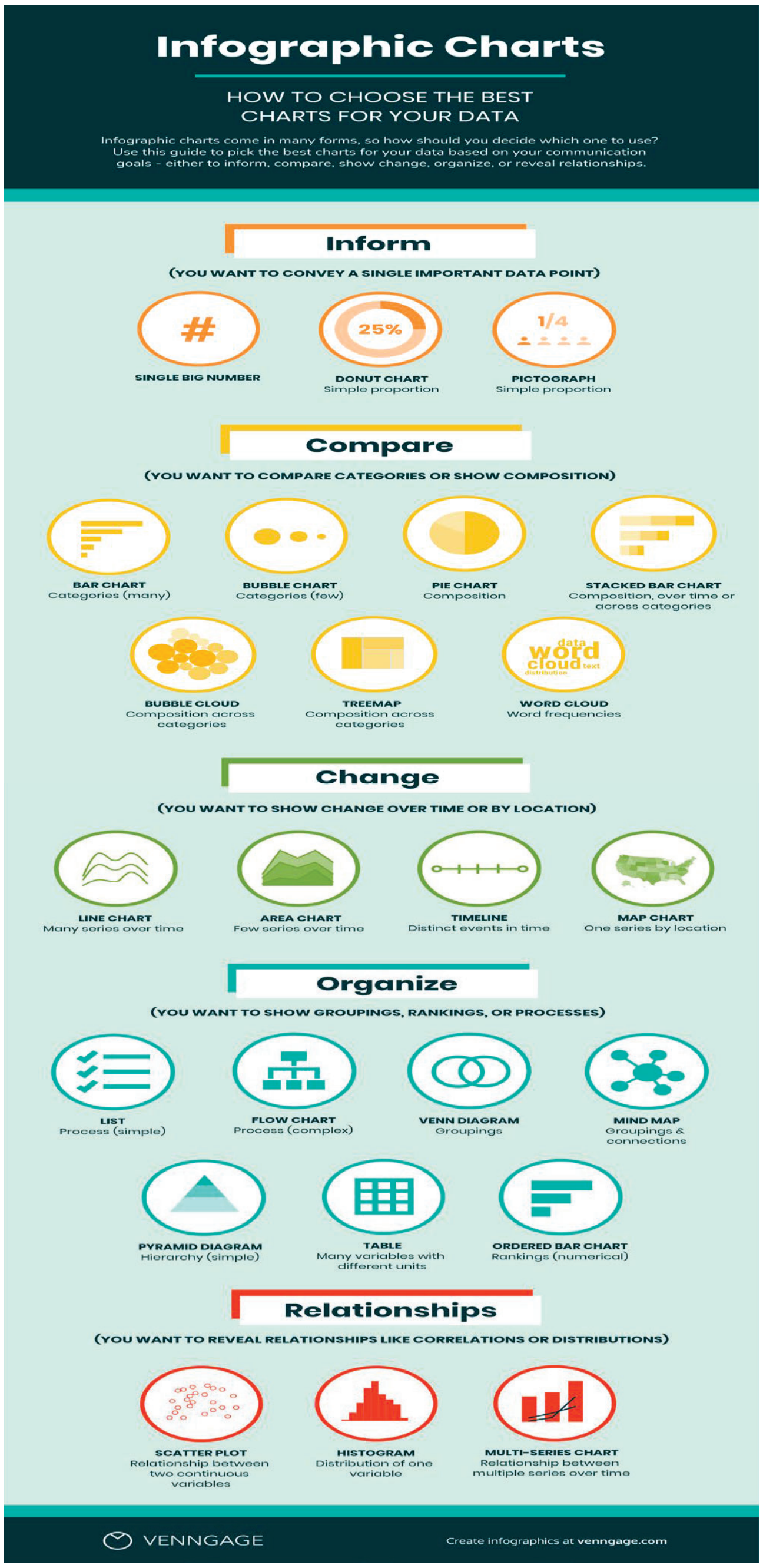

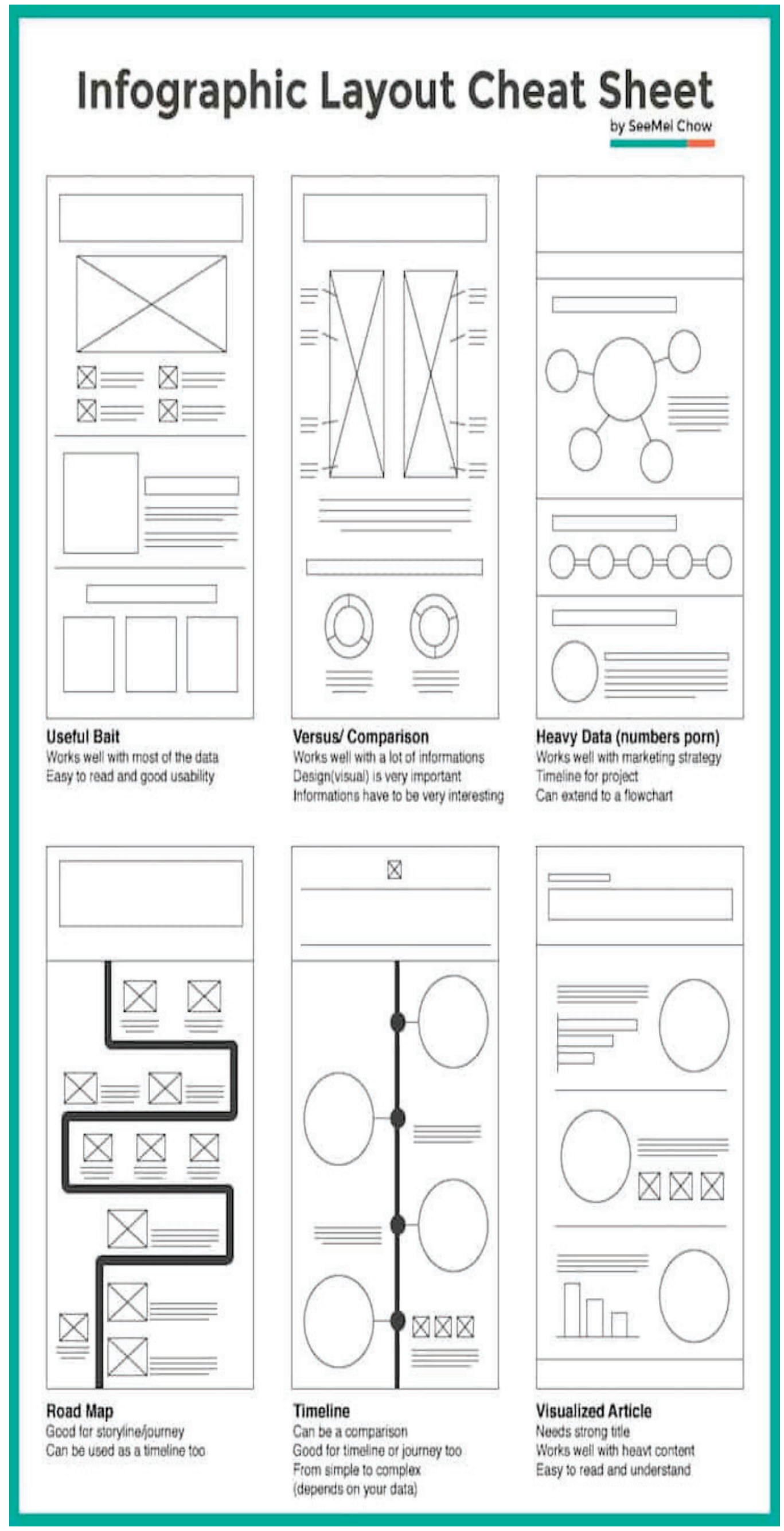

E. Consider fonts. Font type and size convey relevance and tone of your subject and serve as another guide for readers. There are no absolute rules but here are a few tips:

- **Use a legible font size.** Try a 14 or 16 size font so readers do not have to strain to see your content. If this size makes your text too long, try to reduce the text before your shrink the font.
- **Avoid overly stylized fonts for text.** Choose classic fonts that are readable (e.g., Georgia, Times, Arial, Helvetica, and Calibri), and feel free to pair different fonts in headings and subheadings (see chart below).
- **Use high-contrast colors for fonts and visuals.** For fonts and visuals conveying information, use colors that have high contrast together.
- **Avoid white text on dark backgrounds.** White text on dark backgrounds can strain eyes. Ideally, text should be a darker font on a lighter background. If light text is needed/preferred, use a light color (e.g., muted gray).

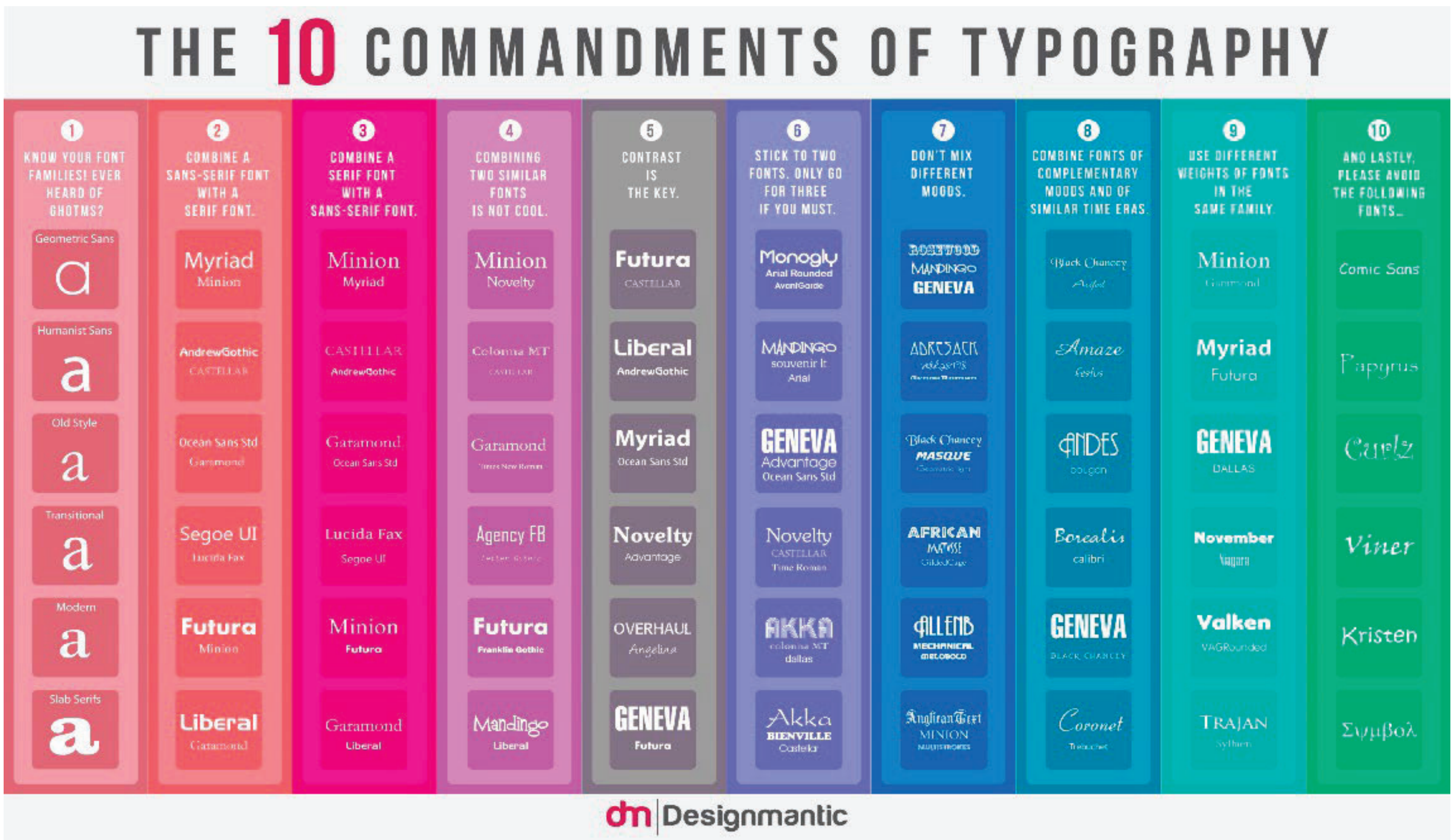

F. Consider Colors.

Colors are symbols and present your research in a particular light. Choose a color palette that fits the theme and tone of the infographic and that is neutral enough for a broader audience. This infographic will be posted/published online so consider colors that read well in this format. Avoid dominant dark and/or neon colors. These are harder to read online and are less preferred for infographics. Be careful about a white background given many websites will have a white background. Bring a subtle contrast to the website and your infographic whenever possible.

A good color rule is the **rule of three**—choose three colors: 1) background color (usually the lightest of the three) and 2) two colors used to break up content and sections. If you *need* to add other colors, use shades of the three main colors to stay cohesive. Lots of tools to select colors can be found as part of the design site (e.g., Canva, etc.) or on free sites such as COLOURlovers or Coolors . Here are some good and bad palettes for infographics:

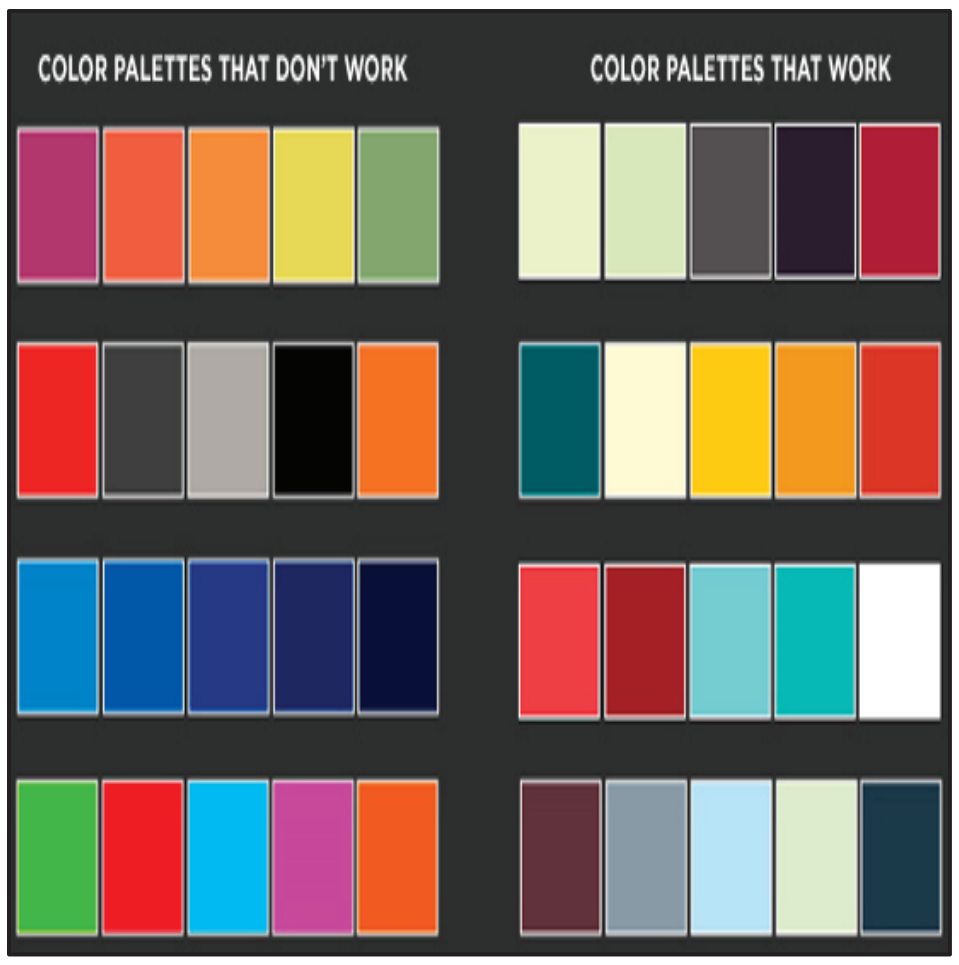

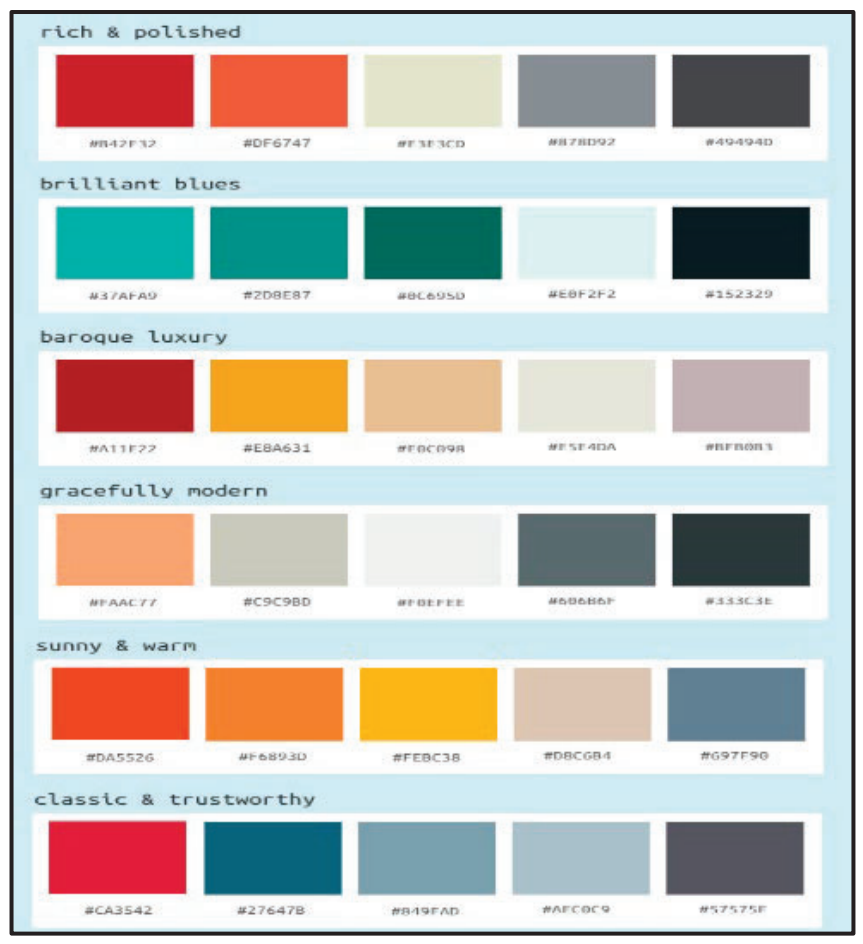

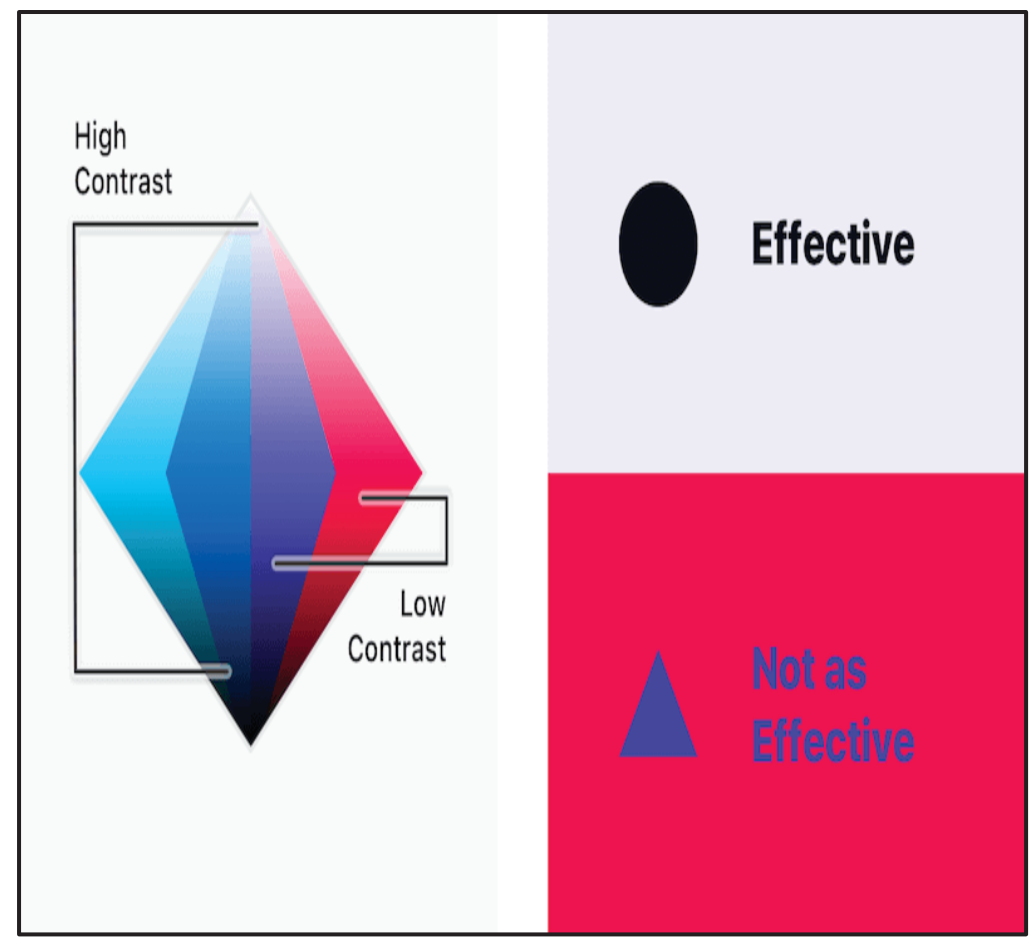

https://www.smashingmagazine.com/2011/10/the-dos-and-donts-of-infographic-design/
Color Palette Infographic Template (venngage.com)

G. Consider white space.

White space is the unused empty or "negative space" on your infographic. It is empty and can be white or the color of your background. Keep white space! It serves multiples purposes: 1) improves readability; 2) reduces overload to a reader; 3) promotes order and flow (for greater understanding of content); and 4) serves to spotlight or highlight central content. See ease.ly for more on white space.
https://www.easel.ly/blog/use-white-space-infographic-design/#:~:text=White%20space%2C%20also%20known%20as%20negative%20space%2C%20is,also%20take%20the%20color%20of%20your%20infographic%E2%80%99s%20background.

H. Consider visuals.

The visual content tells your story so choose wisely. If an icon, chart, or other "clip art" is distracting or irrelevant to the research interpretation and story, get rid of it. Keep visuals that are essential and convey meaning. Check your visual readability. Desaturate each image *by* viewing it in grayscale. If you cannot tell the grays apart, then the original colors don't have high contrast. Review some of your visual options below. Consider your five data points as you plan, select, create your visuals.

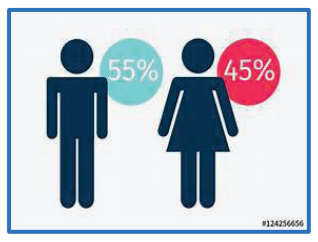

Icons are simple relevant visual symbols that convey a concept in an easy almost universally understandable way. An icon can be coupled with a univariate statistic to inform the topic and findings highlighted in the infographic.

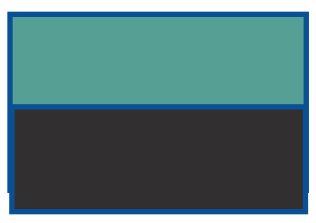

Color blocking is the use of colors to separate content and give a visual cue to readers that the content in distinct. Remember the use of "white space" in color blocking to signal content breaks.

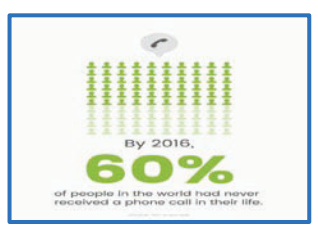

Pictograms are data visualizations of multivariate statistics where each icon represent the units of measurement.

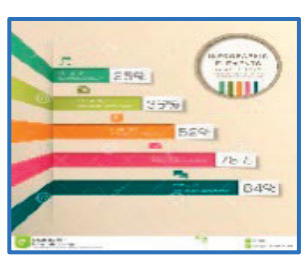

Graphs and charts typically found in the social sciences/public health can be used to convey findings. Pair different types of graphs to separate data findings to provide visual interest. As in a traditional research paper, label your graphs and tables using APA or the numbered IEEE style. You may need to add a brief description to provide context. **You will need to create your own original graph if you chose to include one!**

I. ADA accessibility

As we draft infographics, please keep in mind our responsibility for ADA accessibility. Adding alt text is a great way to achieve ADA accessibility. Alt text is an attribute you can add to any image on your site, including infographics. How al text can be added will vary by your format and site. Cal State offers a great site: https://www.csun.edu/universal-design-center/accessible-infographics-and-flyers-checklist. Other ways to promote accessibility in your infographic include:

- ***Text display***: Text should always be clear and easy to understand for maximum reader comprehension. Avoid using difficult to read font, small text and ensure the language and tone in written text is appropriate to ensure the audience remains engaged.
- ***Links:*** If there are links in an infographic, the link text must clearly describe where the link is going to take a user. Do not use link phrases such as "click here" or "read more" and always ensure the link can be easily distinguished to users.
- ***Color contrast:*** When using color in an infographic, always make sure there is enough color contrast between the foreground content and background content. When the foreground and background colors are too similar, users will have a hard time understanding the information.
- ***Structure:*** an infographic must always have a logical structure that includes titles and heading levels.
- ***Mobile Display:*** Avoid making large infographics as they cannot be properly displayed on mobile devices.

https://www.easel.ly/blog/easelly-accessibility/

J. "Rough cut" or polished rough draft from website

Rough cuts are one step further in development than the sketch and wireframe. A rough cut or polished rough draft offers enough detail to receive feedback for revision. At this stage, your infographic is created within a program (e.g., Canva) and includes font and color choices, some visuals (if not most or all), and text annotation. You may be working on placement, citations, hyperlinks, and even a title. **A rough cut is needed for peer review.**

K. Citations.

Provide APA formatting or the numbered IEEE style formatted citations and ensure that you include accurate links. You will need to use a citation within the text as you use the information (preferred would be number-format citation) and a works-cited as the bottom of the infographic.

L. Peer review

As part of the infographic assignment, we will use multiple peer reviews within our course. This process is meant to be constructive. Peer review serves two purposes: it gives feedback to students on their infographic designs and it supports student reviewers to critically analyze the infographics of others. We will use the criteria below in peer review, and you will give and receive narrative feedback in each category. Feedback can be written or recorded in a video (TBA). Here is a great online tool to assess infographics: https://stephanieevergreen.com/data-visualization-checklist/

Grading and Assessment for Infographics (Matrix & Hodson, 2014; Schrock, 2012)	
Content	Accurate and detailed information is provided and supports the thesis/argument/purpose.
Focus	All content (visual and textual) concisely supports the purpose of the infographic.
Organization	Information is systematically organized and supports the reader's comprehension of the main message.
Visual Appeal	Fonts, colors, layouts, and visual elements meaningfully contribute to the Infographics' overall message.
Argument/Purpose	The Infographic effectively informs and convinces the reader of its intended purpose or thesis.
Citation	Full bibliographic citations are included for all sources referenced. APA used in SC4383.
Mechanics	The infographic is free of spelling and grammatical errors.

M. Publish on StMU Research Scholars

Gillicano and colleagues (2014) suggest that an infographic is easily comprehended and read in less than a minute. Much like an essay, an infographic is often the end product of a research project or inquiry process. Overall, thought should be given to who the target audience is and what purpose the infographic will serve.

Each student will have the opportunity to create and revise an infographic to meet the standards for publication with ***StMU Research Scholars.*** A completed infographic including participation in peer review is required for publication consideration. ***Publication is worth 10 points on the final grade.***

Please visit the site here: https://stmuscholars.org/

This would count as a publication on your resume or CV! This should be a project and product that you are proud of!

Resources

Books

The Functional Art: An Introduction to Information Graphics and Visualization
Data + Design, a simple introduction to preparing and visualizing information

Articles

Emotional Data Visualization: Periscopic's "U.S. Gun Deaths" and the Challenge of Uncertainty
https://www.peachpit.com/articles/article.aspx?p=2036558
Infographics and visualizations as tools for the mind Alberto Cairo
Learning to See Oliver Reichenstein
Visual encoding Michael Dubakov
The Trifecta Checkup framework for visualization criticism Kaiser Fung
An Economist's Guide to Visualizing Data Jon Schwabish
Visual literacy in the age of data Shazna Nessa
Storytelling with data Jonathan Corum

Podcasts and blogs

What makes a good data visualization? With Manuel Lima, Kaiser Fung, Jonathan Stray, and others
http://www.thefunctionalart.com
http://visualisingdata.com/
http://www.perceptualedge.com/blog/
http://www.storytellingwithdata.com/
http://blog.visual.ly/
http://blogs.forbes.com/naomirobbins/

BIO/BL 3310: Introduction to Bioinformatics
Draft Feedback Rubric

Infographic Title:

	5	4	3	2	1	Points Awarded
**Content and Accuracy**<br>• Are the five key points clear, accurate, and supported by credible sources?<br>• Is at least one numeric data point included?	Information is factually accurate, comprehensive, and directly supports the research purpose	Minor factual errors or omissions, otherwise accurate and well-supported.	Some inaccuracies OR insufficient detail in key points.	Several inaccuracies; main points only partially supported.	Content is mostly inaccurate or unsupported.	
**Written Feedback for Content and Accuracy:**						
**Organization & Storytelling**<br>• Does the infographic have a strong title (<12 words)?<br>• Does the "so what" factor or takeaway come across clearly?	Narrative arc is logical and engaging; audience can easily follow the story.	Organization is clear; minor issues with flow or clarity.	Some disjointed sections; story is present but not seamless.	Organization weak; message is hard to follow.	No clear story or flow.	
**Written Feedback for Organization and Storytelling:**						
**Design & Visual Appeal**<br>• Is font size legible (≈14–16)?<br>• Are colors accessible (high contrast, rule of three)?<br>• Do visuals clarify rather than distract?	Effective use of fonts, colors, icons, charts, and white space; visuals enhance meaning.	Strong design; minor improvements could increase readability or impact.	Adequate design; visuals sometimes cluttered or less effective.	Poor design choices reduce readability or clarity.	Distracting or ineffective design; hard to read.	
**Written Feedback for Design and Visual Appeal:**						

	5	4	3	2	1	Points Awarded
**Sources & Citations**<br>• Are citations included on the infographic itself?<br>• Is the works-cited list accurate and complete?	All data clearly cited; references complete and accurate.	Minor errors in formatting or missing details.	Some missing citations or inconsistent style.	Several sources uncited or inaccurately reported.	Few or no sources cited.	
**Written Feedback for Sources and Citations:**						
**Mechanics & Professionalism**<br>• Is the text concise and error-free?<br>• Would you be proud to share this publicly?	Free of grammar/spelling errors; polished and professional.	1–2 minor errors; does not distract from meaning.	Several noticeable errors; somewhat distracting.	Many errors interfere with clarity.	Frequent errors; unprofessional presentation.	
**Written Feedback for Mechanics and Professionalism:**						
					**Total Points Awarded**	
**General Comments and Feedback:**						

BL 2311 – Food and Nutrition
Infographic Project Instructions

Step-by-step guide for BL 2311 – Food and Nutrition
In BL 2311 for your research project, you will create an infographic and write a research outline instead of the traditional original research paper to communicate your research findings. While the traditional original research format is suitable for many different fields and types of studies, this is not the only way to present research and is not always the preferred format for a variety of reasons (e.g., cost and/or obscurity of journal, timing issues with sensitive data, excessive jargon that deters public connection and consumption, audience, etc.). In this infographic project, you are compiling secondary data findings into an analysis to be visually presented as a combination of numbers, text, and images. This infographic will convey research with a clear emphasis on purpose, design, readability, and types of visual representation (e.g., picture, graph, chart, etc.). Your goal is to produce an infographic that: 1) allows a reader to derive the scholarly meaning from it; 2) is sound with meaning and integrity; and 3) is aesthetically appealing (Lankow, Ritchie, & Crooks, 2012).

Additionally, it is important to be able to relay important information quickly in an easy-to-understand format. For this project, you will convey, through an infographic, a topic of your choice surrounding food and nutrition. Examples of concentrations (you are not limited to this list):

- Diets and/or diet fads
- Diseases related to food and nutrition
- How the body uses certain nutrients / food groups
- How diets have changed over time
- What are nutritional needs at different life stages

There are three key objectives to this project:

A. Translating key empirical finding into a visually communicated message that includes graphs or charts, a narrative, descriptive text, icons and/or images.
B. Developing digital proficiency in infographics in both content creation and visual understanding (e.g., use of color and white space, text boxes, font sizes, placement of text, universal design, etc.).
C. Demonstrating an ability to use technology as a form of research storytelling.

Goals assessment:

1. Evaluation of the ability to use library refereed sources to collect public health data and organize, evaluate, and select data that is appropriate for the infographic.
2. Evaluation of the design, coherence, and readability of the infographic.
3. Encouragement to share the infographic with other publics and individuals outside the university through StMU Research Scholars.

Your final infographic will consist of text annotation and visualizations that convey your selected research topic framed by scholarly data. You will create a one-page infographic that **includes five key points** with at least **one numeric data point**. You may use Canva (www.canva.com - there are free educational accounts!) or other similar website to create your infographic using a presentation or infographic template. If you want to make an interactive infographic, you can Genially also has free templates that you can use (https://genially.com/create/infographics/). Well-executed interactive infographics may earn bonus points on the assignment (not guaranteed, this is left to instructor discretion).
The following is a guide to the development and successful completion of your infographic. Space and/or text boxes are provided for you to fill in your work as it develops.

- Infographics need to be 1600 – 1700 pixels wide to display at their fullest.
  - The "resize" setting is in the upper left corner of Canva.
  - For formatting, portrait orientation is preferred

A. Title of Infographic (*less than 12 words, descriptive, interesting to readers, accurate to content)*

B. The story. What is the story you are telling? The story should dictate the design and reflect the research purpose (e.g., descriptive, exploratory, explanatory, evaluation). Use the following questions to get at your data viz story: *What story am I telling? What do I already know about it and what will I need to research? Where will I get that information? Why does this story need to be told? Who will be my audience? What does my audience need to know and how will they want to learn it? What do you want your audience to **do** with this information?*

C. Central data points/annotations.

What is the order of your key points? *(think narrative arc)*

What is the summarizing/concluding statement (the "so what" factor)?

What is the central hook or take-away? "Hero" statistic? Where will you visually locate this hook (e.g., center, at the end)?

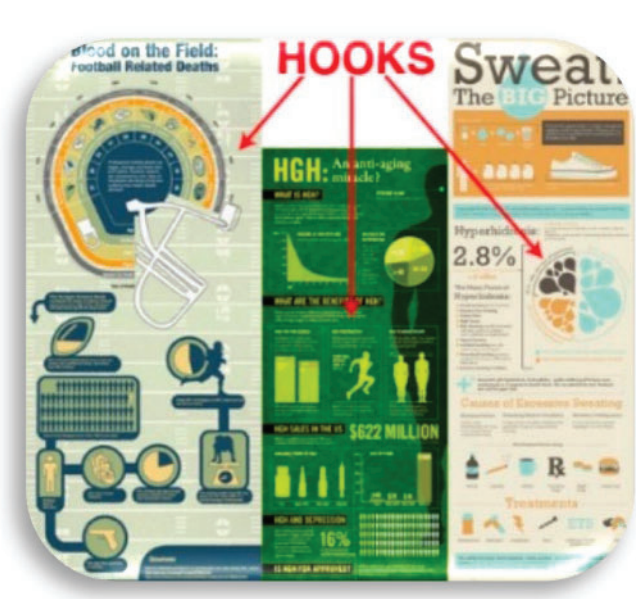

D. Additional scholarly and non-scholarly references. Use APA the numbered IEEE style format.

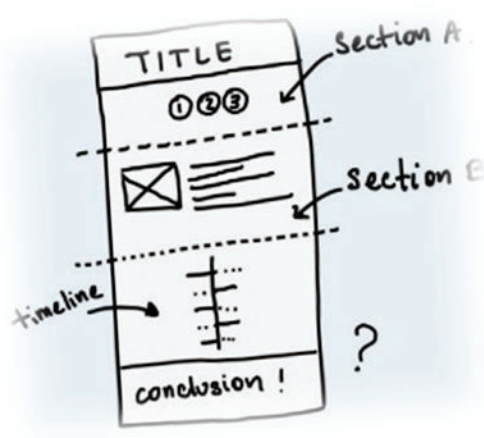

- **Infographic Sketch also referred to as "a wireframe" or a visual representation that demonstrates the relationship of elements on a page. Select draft layout, text, and visuals.**
- 9 x 11" standard size (saved as jpeg or PDF)—vertical or horizontal
- *Before you sketch, consider type of visual representation (process, flow, comparison, timeline, etc.) that would make the most sense for conveying the information needed.*

Example of an infographic; the Texas Medical Association has some great examples! www.texmed.org

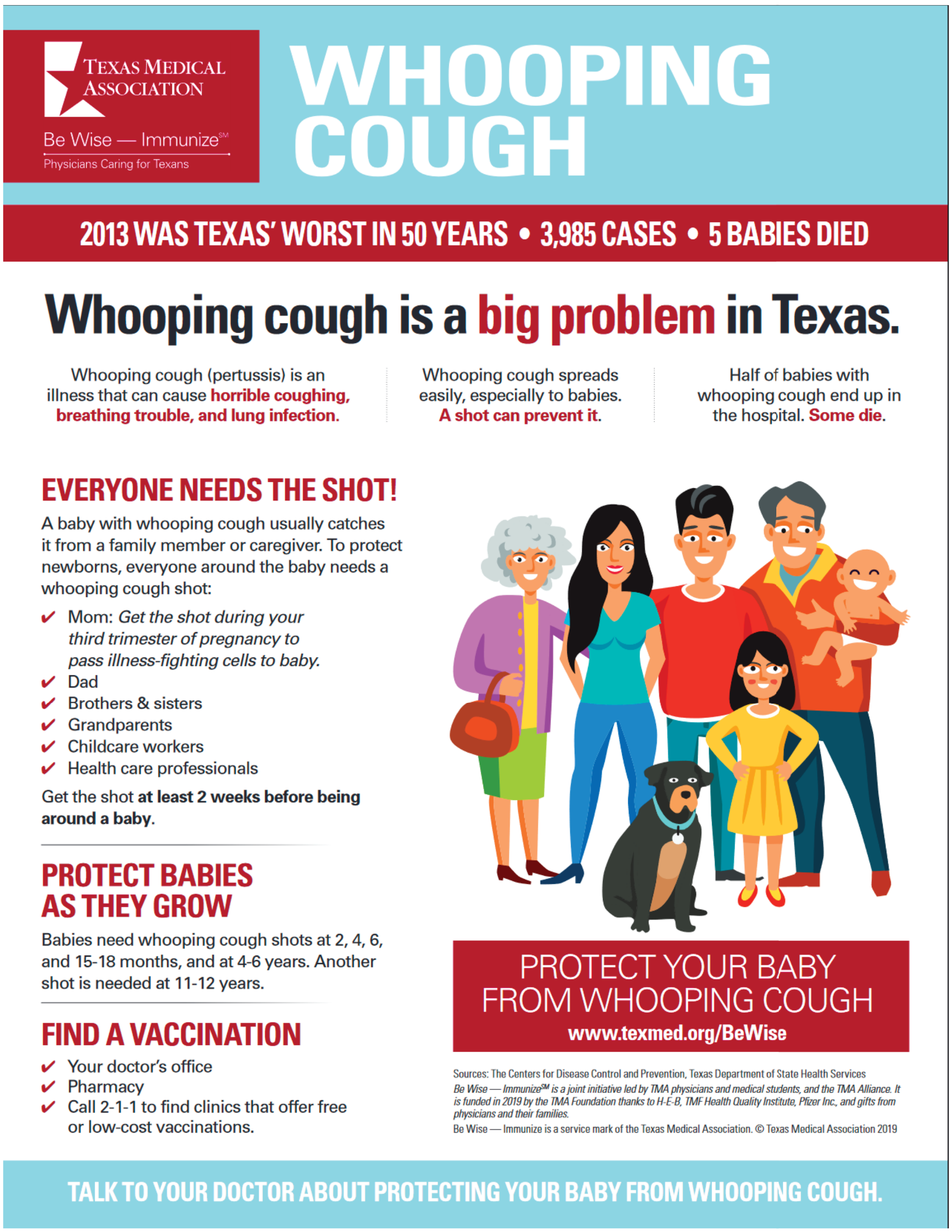

Here are two resources for you to consider as you create a layout and choose charts, graphics, and visuals.

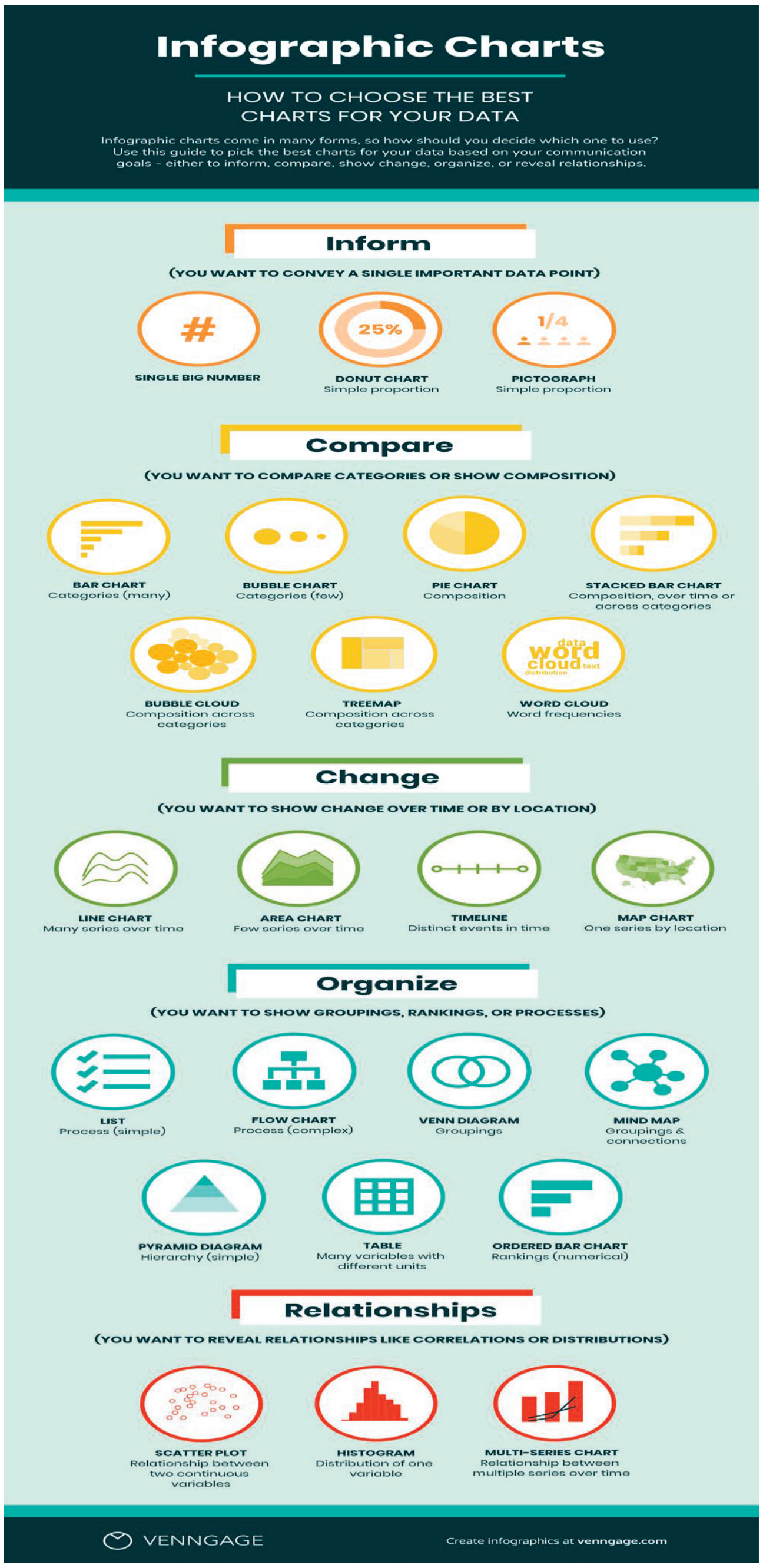

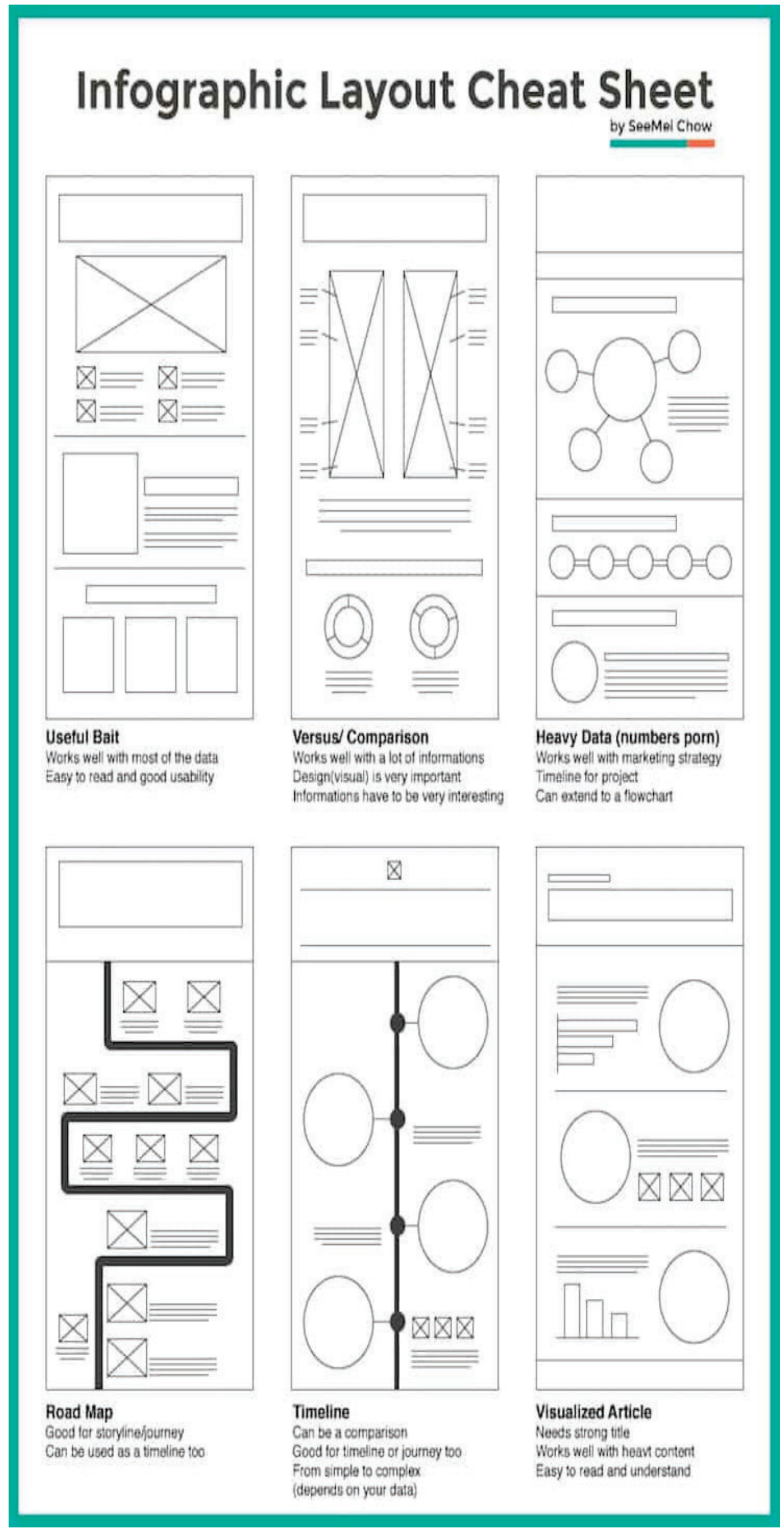

E. **Consider fonts.** Font type and size convey relevance and tone of your subject and serve as another guide for readers. There are no absolute rules but here are a few tips:
   - **Use a legible font size.** Try a 14 or 16 size font so readers do not have to strain to see your content. If this size makes your text too long, try to reduce the text before your shrink the font.
   - **Avoid overly stylized fonts for text.** Choose classic fonts that are readable (e.g., Georgia, Times, Arial, Helvetica, and Calibri), and feel free to pair different fonts in headings and subheadings (see chart below).
   - **Use high-contrast colors for fonts and visuals.** For fonts and visuals conveying information, use colors that have high contrast together.
   - **Avoid white text on dark backgrounds.** White text on dark backgrounds can strain eyes. Ideally, text should be a darker font on a lighter background. If light text is needed/preferred, use a light color (e.g., muted gray).

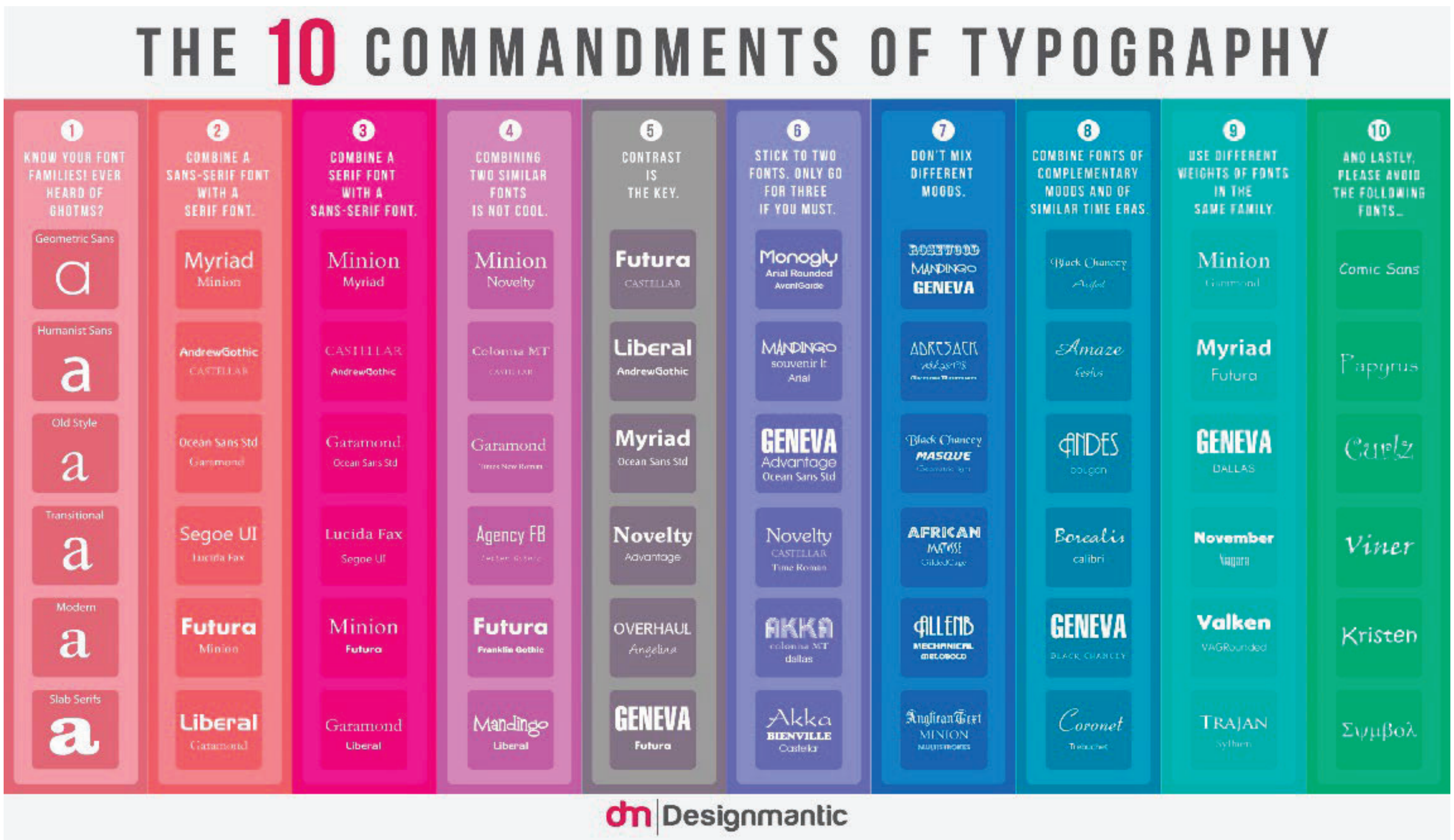

F. Consider Colors.

Colors are symbols and present your research in a particular light. Choose a color palette that fits the theme and tone of the infographic and that is neutral enough for a broader audience. This infographic will be posted/published online so consider colors that read well in this format. Avoid dominant dark and/or neon colors. These are harder to read online and are less preferred for infographics. Be careful about a white background given many websites will have a white background. Bring a subtle contrast to the website and your infographic whenever possible.

A good color rule is the **rule of three**—choose three colors: 1) background color (usually the lightest of the three) and 2) two colors used to break up content and sections. If you *need* to add other colors, use shades of the three main colors to stay cohesive. Lots of tools to select colors can be found as part of the design site (e.g., Canva, etc.) or on free sites such as COLOURlovers or Coolors . Here are some good and bad palettes for infographics:

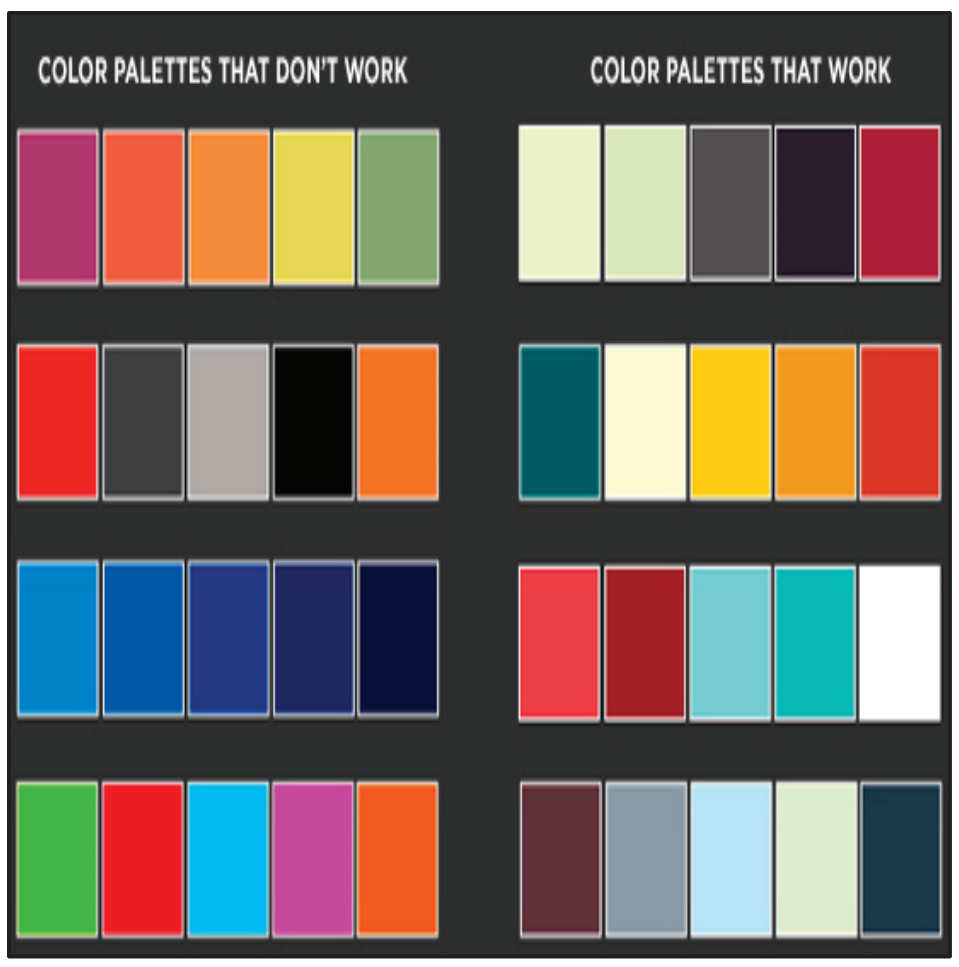

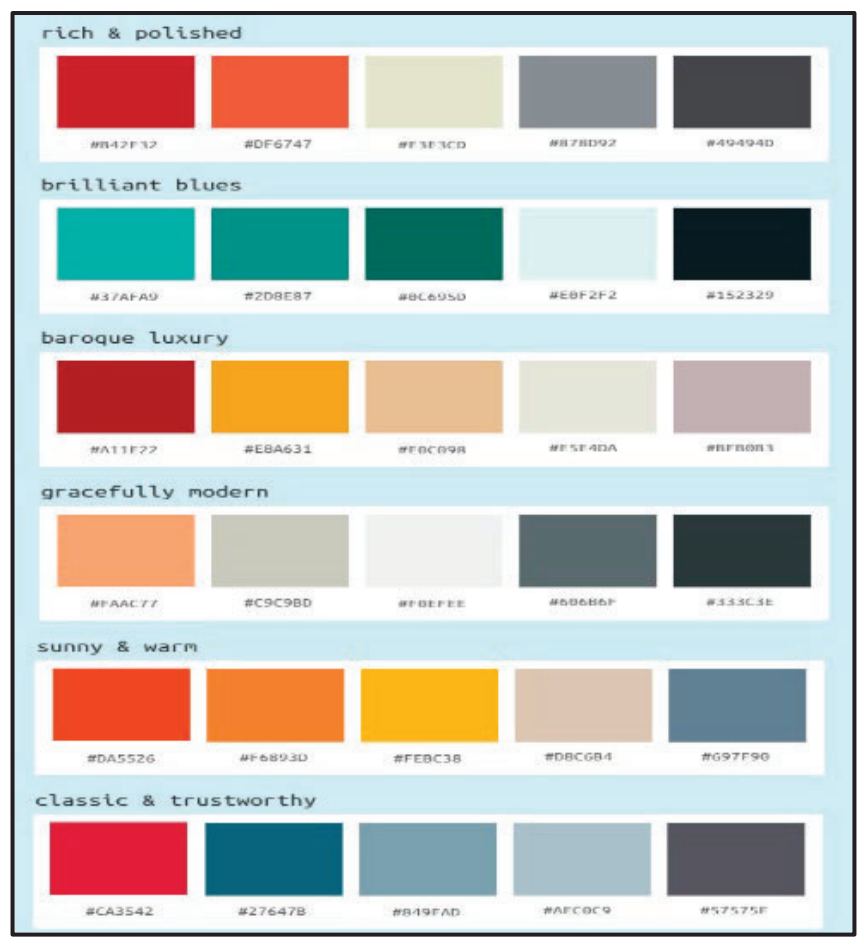

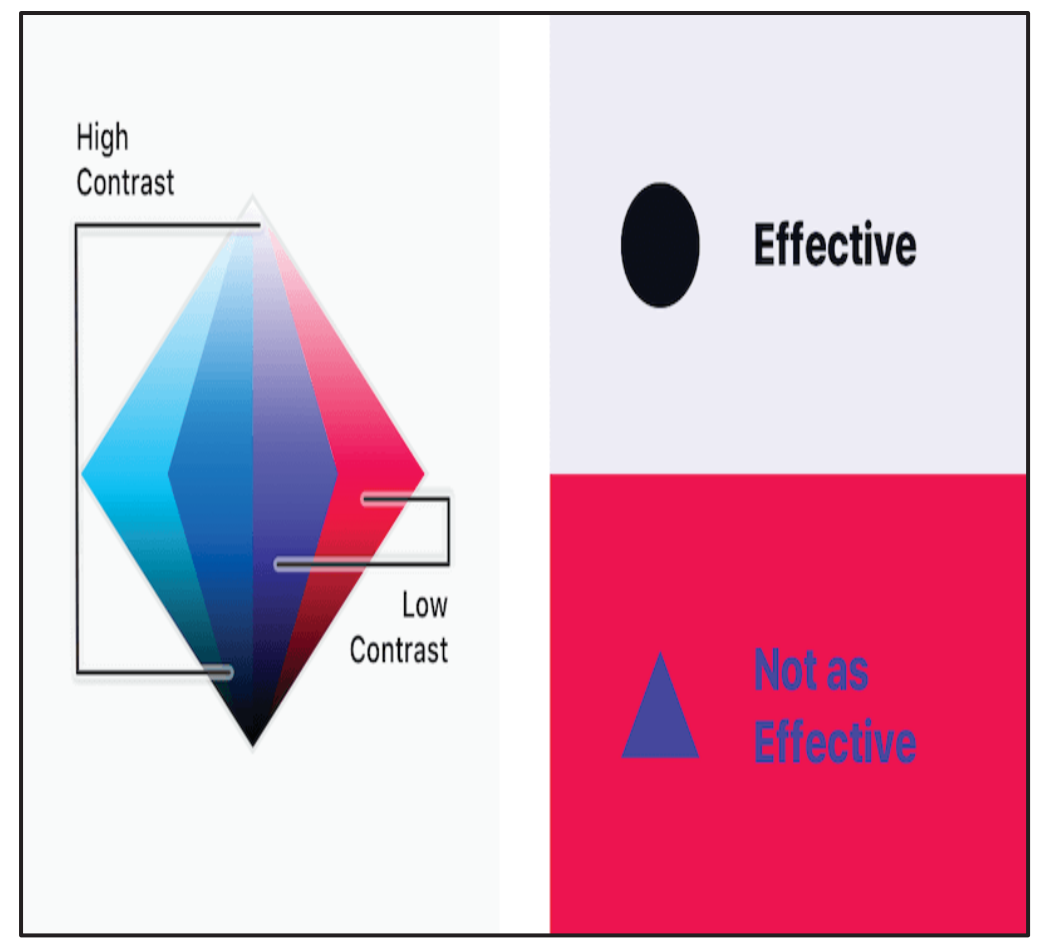

https://www.smashingmagazine.com/2011/10/the-dos-and-donts-of-infographic-design/
Color Palette Infographic Template (venngage.com)

G. Consider white space.

White space is the unused empty or "negative space" on your infographic. It is empty and can be white or the color of your background. Keep white space! It serves multiples purposes: 1) improves readability; 2) reduces overload to a reader; 3) promotes order and flow (for greater understanding of content); and 4) serves to spotlight or highlight central content. See ease.ly for more on white space.
https://www.easel.ly/blog/use-white-space-infographic-design/#:~:text=White%20space%2C%20also%20known%20as%20negative%20space%2C%20is,also%20take%20the%20color%20of%20your%20infographic%E2%80%99s%20background.

H. Consider visuals.

The visual content tells your story so choose wisely. If an icon, chart, or other "clip art" is distracting or irrelevant to the research interpretation and story, get rid of it. Keep visuals that are essential and convey meaning. Check your visual readability. Desaturate each image *by* viewing it in grayscale. If you cannot tell the grays apart, then the original colors don't have high contrast. Review some of your visual options below. Consider your five data points as you plan, select, create your visuals.

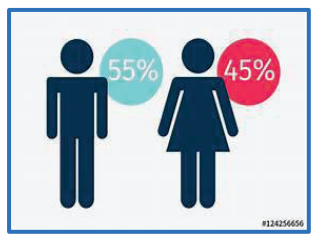

Icons are simple relevant visual symbols that convey a concept in an easy almost universally understandable way. An icon can be coupled with a univariate statistic to inform the topic and findings highlighted in the infographic.

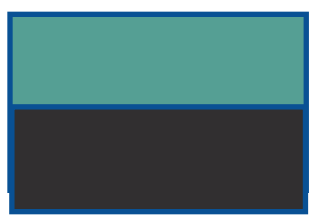

Color blocking is the use of colors to separate content and give a visual cue to readers that the content in distinct. Remember the use of "white space" in color blocking to signal content breaks.

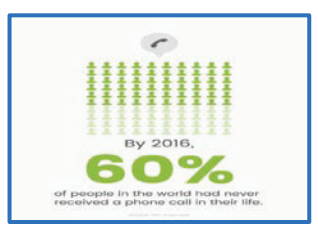

Pictograms are data visualizations of multivariate statistics where each icon represent the units of measurement.

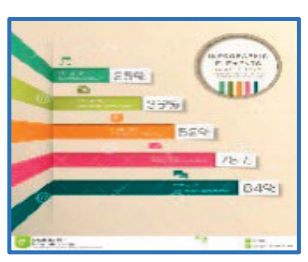

Graphs and charts typically found in the social sciences/public health can be used to convey findings. Pair different types of graphs to separate data findings to provide visual interest. As in a traditional research paper, label your graphs and tables using APA or the numbered IEEE style. You may need to add a brief description to provide context. **You will need to create your own original graph if you chose to include one!**

I. ADA accessibility

As we draft infographics, please keep in mind our responsibility for ADA accessibility. Adding alt text is a great way to achieve ADA accessibility. Alt text is an attribute you can add to any image on your site, including infographics. How al text can be added will vary by your format and site. Cal State offers a great site: https://www.csun.edu/universal-design-center/accessible-infographics-and-flyers-checklist. Other ways to promote accessibility in your infographic include:

- ***Text display***: Text should always be clear and easy to understand for maximum reader comprehension. Avoid using difficult to read font, small text and ensure the language and tone in written text is appropriate to ensure the audience remains engaged.
- ***Links:*** If there are links in an infographic, the link text must clearly describe where the link is going to take a user. Do not use link phrases such as "click here" or "read more" and always ensure the link can be easily distinguished to users.
- ***Color contrast:*** When using color in an infographic, always make sure there is enough color contrast between the foreground content and background content. When the foreground and background colors are too similar, users will have a hard time understanding the information.
- ***Structure:*** an infographic must always have a logical structure that includes titles and heading levels.
- ***Mobile Display:*** Avoid making large infographics as they cannot be properly displayed on mobile devices.

https://www.easel.ly/blog/easelly-accessibility/

J. "Rough cut" or polished rough draft from website

Rough cuts are one step further in development than the sketch and wireframe. A rough cut or polished rough draft offers enough detail to receive feedback for revision. At this stage, your infographic is created within a program (e.g., Canva) and includes font and color choices, some visuals (if not most or all), and text annotation. You may be working on placement, citations, hyperlinks, and even a title. **A rough cut is needed for peer review.**

K. Citations.

Provide APA formatting or the numbered IEEE style formatted citations and ensure that you include accurate links. You will need to use a citation within the text as you use the information (preferred would be number-format citation) and a works-cited as the bottom of the infographic.

L. Peer review

As part of the infographic assignment, we will use multiple peer reviews within our course. This process is meant to be constructive. Peer review serves two purposes: it gives feedback to students on their infographic designs and it supports student reviewers to critically analyze the infographics of others. We will use the criteria below in peer review, and you will give and receive narrative feedback in each category. Feedback can be written or recorded in a video (TBA). Here is a great online tool to assess infographics: https://stephanieevergreen.com/data-visualization-checklist/

Grading and Assessment for Infographics (Matrix & Hodson, 2014; Schrock, 2012)	
Content	Accurate and detailed information is provided and supports the thesis/argument/purpose.
Focus	All content (visual and textual) concisely supports the purpose of the infographic.
Organization	Information is systematically organized and supports the reader's comprehension of the main message.
Visual Appeal	Fonts, colors, layouts, and visual elements meaningfully contribute to the Infographics' overall message.
Argument/Purpose	The Infographic effectively informs and convinces the reader of its intended purpose or thesis.
Citation	Full bibliographic citations are included for all sources referenced. APA used in SC4383.
Mechanics	The infographic is free of spelling and grammatical errors.

M. Publish on StMU Research Scholars

Gillicano and colleagues (2014) suggest that an infographic is easily comprehended and read in less than a minute. Much like an essay, an infographic is often the end product of a research project or inquiry process. Overall, thought should be given to who the target audience is and what purpose the infographic will serve.

Each student will have the opportunity to create and revise an infographic to meet the standards for publication with ***StMU Research Scholars.*** A completed infographic including participation in peer review is required for publication consideration. ***Publication is worth 10 points on the final grade.***

Please visit the site here: https://stmuscholars.org/

This would count as a publication on your resume or CV! This should be a project and product that you are proud of!

Resources

Books

The Functional Art: An Introduction to Information Graphics and Visualization
Data + Design, a simple introduction to preparing and visualizing information

Articles

Emotional Data Visualization: Periscopic's "U.S. Gun Deaths" and the Challenge of Uncertainty
https://www.peachpit.com/articles/article.aspx?p=2036558
Infographics and visualizations as tools for the mind Alberto Cairo
Learning to See Oliver Reichenstein
Visual encoding Michael Dubakov
The Trifecta Checkup framework for visualization criticism Kaiser Fung
An Economist's Guide to Visualizing Data Jon Schwabish
Visual literacy in the age of data Shazna Nessa
Storytelling with data Jonathan Corum

Podcasts and blogs

What makes a good data visualization? With Manuel Lima, Kaiser Fung, Jonathan Stray, and others
http://www.thefunctionalart.com
http://visualisingdata.com/
http://www.perceptualedge.com/blog/
http://www.storytellingwithdata.com/
http://blog.visual.ly/
http://blogs.forbes.com/naomirobbins/

BL 2311 – Food and Nutrition
Draft Feedback Rubric

Infographic Title:

	5	4	3	2	1	Points Awarded
**Content and Accuracy**<br>• Are the five key points clear, accurate, and supported by credible sources?<br>• Is at least one numeric data point included?	Information is factually accurate, comprehensive, and directly supports the research purpose	Minor factual errors or omissions, otherwise accurate and well-supported.	Some inaccuracies OR insufficient detail in key points.	Several inaccuracies; main points only partially supported.	Content is mostly inaccurate or unsupported.	
**Written Feedback for Content and Accuracy:**						
**Organization & Storytelling**<br>• Does the infographic have a strong title (<12 words)?<br>• Does the "so what" factor or takeaway come across clearly?	Narrative arc is logical and engaging; audience can easily follow the story.	Organization is clear; minor issues with flow or clarity.	Some disjointed sections; story is present but not seamless.	Organization weak; message is hard to follow.	No clear story or flow.	
**Written Feedback for Organization and Storytelling:**						
**Design & Visual Appeal**<br>• Is font size legible (≈14–16)?<br>• Are colors accessible (high contrast, rule of three)?<br>• Do visuals clarify rather than distract?	Effective use of fonts, colors, icons, charts, and white space; visuals enhance meaning.	Strong design; minor improvements could increase readability or impact.	Adequate design; visuals sometimes cluttered or less effective.	Poor design choices reduce readability or clarity.	Distracting or ineffective design; hard to read.	
**Written Feedback for Design and Visual Appeal:**						

	5	4	3	2	1	Points Awarded
**Sources & Citations**<br>• Are citations included on the infographic itself?<br>• Is the works-cited list accurate and complete?	All data clearly cited; references complete and accurate.	Minor errors in formatting or missing details.	Some missing citations or inconsistent style.	Several sources uncited or inaccurately reported.	Few or no sources cited.	
**Written Feedback for Sources and Citations:**						
**Mechanics & Professionalism**<br>• Is the text concise and error-free?<br>• Would you be proud to share this publicly?	Free of grammar/spelling errors; polished and professional.	1–2 minor errors; does not distract from meaning.	Several noticeable errors; somewhat distracting.	Many errors interfere with clarity.	Frequent errors; unprofessional presentation.	
**Written Feedback for Mechanics and Professionalism:**						
					**Total Points Awarded**	
**General Comments and Feedback:**						

Appendix 2: Student Perception Survey

Tools Saved at 6:41 PM Published Preview Publish

AI Feedback Survey

ExpertReview score Fair

Purpose and Consent

Consent

Study Title: Analysis of AI Feedback within the Asynchronous Online STEM Classroom
Researcher: Lori Boies, PhD, MPH St. Mary's University Department of Biological Sciences; lboies@stmarytx.edu; 210-436-3521
IRB: IRB_Committee@stmarytx.edu

Purpose. You are invited to take part in a research study about how instructor, peer, and AI-generated feedback support semester-long projects in BL 2311, BL 3310, and BL 4411. Study may also compare publicly posted ((https://stmuscholars.org/) student projects from Fall 2025 with prior years.

Procedures. If you agree, you will complete an online survey (~10–12 minutes) about your experiences using feedback and your project revisions. You may skip any question or stop at any time.

Risks/Discomforts. Minimal risk (possible minor discomfort reflecting on your work). Privacy risks are mitigated by de-identification and secure data handling.

Benefits. There may be no direct benefit to you. Findings may improve feedback practices and course design.

Confidentiality. Responses are de-identified and reported in aggregate. Names and grades are not collected. IP address collection in Qualtrics is disabled. Publicly posted course artifacts may be analyzed separately. Data are stored securely and accessed only by the research team.

Voluntary Participation. Your participation is voluntary and anonymous and will not affect your grade or standing. There will be no way for Dr. Boies to see the answers of a particular student.l

Contacts. For questions about the study, contact Dr. Lori Boies (lboies@stmarytx.edu or 210-436-3521).

If you have any questions about your rights as a research subject or concerns about this research study please contact the Chair, Institutional Review Board, St. Mary's University at 210-436-3736 or email at IRBCommitteeChair@stmarytx.edu.

Consent. By selecting “I agree” below, you confirm you are 18 years or older, have read this information, and voluntarily consent to participate.

- ◯ I agree to participate
- ◯ I do not agree to participate

Appendix 2: Student Perception Survey

Import from library
Add new question

Add Block

Background and Access

Q4

Please Select Your Course

- BL 2311: Food and Nutrition
- BL/BIO 3310: Introduction to Bioinformatics
- BL/BIO 4411: Genes, Genomes, and Genomics

Q5

What is your major?

Q6

What is your class standing?

- 1st Year (Freshman)
- 2nd Year (Sophomore)
- 3rd Year (Junior)
- 4th Year (Senior)
- 4+

Q7

Prior experience using AI tools for schoolwork?

- None
- A little
- Moderate
- A lot

Q8

Typical device for coursework

- Laptop
- Desktop
- Tablet
- Phone
- Mixed

Q9

Reliable internet access for course work?

- Never
- Sometimes
- About half the time
- Most of the time
- Always

Import from library Add new question

Add Block

Project and Revision Process

Q12

Which of the following projects were you assigned?

- Academic Explanatory Article
- Infographic

Q10

I completed all major checkpoints (proposal, outline, draft, final)

- Yes
- No

Appendix 2: Student Perception Survey

Q11

Number of revision cycles you completed for the paper

- ◯ 1
- ◯ 2
- ◯ 3
- ◯ 4+

Q13

Did you publish your final product on the StMU Research Scholars website?

- ◯ Yes
- ◯ No

Import from library

Add new question

Add Block

Usefulness of Feedback

Q14

Think about each of the comments/feedback you received throughout the study, and rate how useful it was for the following question:
Helped me identify weaknesses

	Not Useful	Slightly Useful	Moderately Useful	Very Useful	Extremely Useful
Peer Feedback	◯	◯	◯	◯	◯
Instructor Feedback	◯	◯	◯	◯	◯
AI Feedback	◯	◯	◯	◯	◯

Q15

Think about each of the comments/feedback you received throughout the study, and rate how useful it was for the following question:
Was specific and actionable

	Not Useful	Slightly Useful	Moderately Useful	Very Useful	Extremely Useful
Peer Feedback	○	○	○	○	○
Instructor Feedback	○	○	○	○	○
AI Feedback	○	○	○	○	○

Q16

Think about each of the comments/feedback you received throughout the study, and rate how useful it was for the following question:
Was timely enough to use

	Not Useful	Slightly Useful	Moderately Useful	Very Useful	Extremely Useful
Peer Feedback	○	○	○	○	○
Instructor Feedback	○	○	○	○	○
AI Feedback	○	○	○	○	○

Q17

Think about each of the comments/feedback you received throughout the study, and rate how useful it was for the following question:
Improved organization and clarity

	Not Useful	Slightly Useful	Moderately Useful	Very Useful	Extremely Useful
Peer Feedback	○	○	○	○	○
Instructor Feedback	○	○	○	○	○
AI Feedback	○	○	○	○	○

Q18

Think about each of the comments/feedback you received throughout the study, and rate how useful it was for the following question:
Improved accuracy of scientific content.

	Not Useful	Slightly Useful	Moderately Useful	Very Useful	Extremely Useful
Peer Feedback	○	○	○	○	○
Instructor Feedback	○	○	○	○	○
AI Feedback	○	○	○	○	○

Q19

Think about each of the comments/feedback you received throughout the study and answer the following question:
I incorporated the feedback into the next draft of my product.

	Never	Rarely	Sometimes	Often	Always
Peer Feedback	○	○	○	○	○
Instructor Feedback	○	○	○	○	○
AI Feedback	○	○	○	○	○

Import from library | Add new question

Add Block

Trust, Clarity, and Cognitive Effort

Q20

Think about each of the comments/feedback you received throughout the study and answer the following question:
I trusted this source of feedback.

	Never	Rarely	Sometimes	Often	Always
Peer Feedback	○	○	○	○	○
Instructor Feedback	○	○	○	○	○
AI Feedback	○	○	○	○	○

Q21

Think about each of the comments/feedback you received throughout the study and answer the following question:
I understood why the feedback suggested changes.

	Never	Rarely	Sometimes	Often	Always
Peer Feedback	○	○	○	○	○
Instructor Feedback	○	○	○	○	○
AI Feedback	○	○	○	○	○

Q22

Think about each of the comments/feedback you received throughout the study and answer the following question:
Effort required to apply this feedback:

	Very Hard	Hard	Neither Easy nor Hard	Easy	Very Easy
Peer Feedback	○	○	○	○	○
Instructor Feedback	○	○	○	○	○
AI Feedback	○	○	○	○	○

Import from library   Add new question

Add Block

Actionability & Uptake (AI-specific)

Q23

AI feedback contained concrete examples or rewrites I could adapt.

	Never	Rarely	Sometimes	Often	Always
AI Feedback	○	○	○	○	○

Q24

AI feedback included incorrect or misleading suggestions.

	Never	Rarely	Sometimes	Often	Always
AI Feedback	○	○	○	○	○

Q25

I verified AI-suggested changes with sources or course materials.

	Never	Rarely	Sometimes	Often	Always
AI Feedback	○	○	○	○	○

Q26

I kept a change log noting which AI suggestions I accepted/rejected.

	Never	Rarely	Sometimes	Often	Always
AI Feedback	○	○	○	○	○

Q27

Top reasons I rejected AI suggestions (check all that apply):

- ☐ Inaccurate content
- ☐ Didn't match rubric
- ☐ Unclear
- ☐ Conflicted with other feedback
- ☐ Conflicted with course materials
- ☐ Time
- ☐ Other

Import from library Add new question

Add Block

Perceived Learning and Performance

Q28

My ability to communicate science improved with this project.

- ○ Yes
- ○ Maybe
- ○ No

Q29

My understanding of biology content improved.

- Yes
- Maybe
- No

Q30

The final product (infographic or paper) quality improved because of AI feedback.

- Yes
- Maybe
- No

Q31

Compared to instructor feedback, AI feedback was:

- Much Worse
- Somewhat worse
- About the same
- Somewhat better
- Much better

Q32

Compared to peer feedback, AI feedback was:

- Much Worse
- Somewhat worse
- About the same
- Somewhat better
- Much better

Import from library Add new question

Add Block

Infographic-Specific

AI feedback improved

AI feedback improved accuracy of labels, units, and figure captions.

- Yes
- Maybe
- No

Q34

AI feedback improved visual hierarchy and readability.

- Yes
- Maybe
- No

Q35

AI feedback reduced misleading or exaggerated claims.

- Yes
- Maybe
- No

Import from library | Add new question

Add Block

Academic Integrity & Process

Q36

I clearly cited sources for factual claims added after AI feedback

- Yes
- No

Q37

I can distinguish between AI wording and my own writing in the final submission

- Yes
- No

Q38

I followed course guidelines on appropriate AI use.

- Yes
- No

Q42

How likely are you to use AI in the future for academic work?

- Extremely unlikely
- Somewhat unlikely
- Neither likely nor unlikely
- Somewhat likely
- Extremely likely

Import from library | Add new question

Add Block

Open-Ended Questions

Q39

Describe one AI suggestion you kept and why.

Q40

Describe one AI suggestion you rejected and why

Q41

What change would make AI feedback more useful in this course?

Q42

Anything else you want the instructors/researchers to know?

Import from library | Add new question

Add Block

Permission to Use Responses

Q43

I consent to the use of my de-identified survey data for research and publication

- Yes
- No

Import from library | Add new question

Add Block

End of Survey

We thank you for your time spent taking this survey.

Your response has been recorded.
